# Genetic Basis of Virulence Attenuation Revealed by Comparative Genomic Analysis of *Mycobacterium tuberculosis* Strain H37Ra *versus* H37Rv

**DOI:** 10.1371/journal.pone.0002375

**Published:** 2008-06-11

**Authors:** Huajun Zheng, Liangdong Lu, Bofei Wang, Shiying Pu, Xianglin Zhang, Genfeng Zhu, Wanliang Shi, Lu Zhang, Honghai Wang, Shengyue Wang, Guoping Zhao, Ying Zhang

**Affiliations:** 1 State Key Laboratory of Genetic Engineering, Department of Microbiology, School of Life Sciences, Fudan University, Shanghai, China; 2 Shanghai-MOST Key Laboratory of Health and Disease Genomics, Chinese National Human Genome Center at Shanghai, Shanghai, China; 3 Department of Molecular Microbiology and Immunology, Bloomberg School of Public Health, Johns Hopkins University, Baltimore, Maryland, United States of America; University of Minnesota, United States of America

## Abstract

Tuberculosis, caused by *Mycobacterium tuberculosis*, remains a leading infectious disease despite the availability of chemotherapy and BCG vaccine. The commonly used avirulent *M. tuberculosis* strain H37Ra was derived from virulent strain H37 in 1935 but the basis of virulence attenuation has remained obscure despite numerous studies. We determined the complete genomic sequence of H37Ra ATCC25177 and compared that with its virulent counterpart H37Rv and a clinical isolate CDC1551. The H37Ra genome is highly similar to that of H37Rv with respect to gene content and order but is 8,445 bp larger as a result of 53 insertions and 21 deletions in H37Ra relative to H37Rv. Variations in repetitive sequences such as IS*6110* and PE/PPE/PE-PGRS family genes are responsible for most of the gross genetic changes. A total of 198 single nucleotide variations (SNVs) that are different between H37Ra and H37Rv were identified, yet 119 of them are identical between H37Ra and CDC1551 and 3 are due to H37Rv strain variation, leaving only 76 H37Ra-specific SNVs that affect only 32 genes. The biological impact of missense mutations in protein coding sequences was analyzed *in silico* while nucleotide variations in potential promoter regions of several important genes were verified by quantitative RT-PCR. Mutations affecting transcription factors and/or global metabolic regulations related to *in vitro* survival under aging stress, and mutations affecting cell envelope, primary metabolism, *in vivo* growth as well as variations in the PE/PPE/PE-PGRS family genes, may underlie the basis of virulence attenuation. These findings have implications not only for improved understanding of pathogenesis of *M. tuberculosis* but also for development of new vaccines and new therapeutic agents.

## Introduction

Tuberculosis (TB) remains a leading infectious disease despite the availability of chemotherapy and the BCG vaccine. *Mycobacterium tuberculosis,* the causative agent of TB, is a highly successful pathogen that has latently infected one third of the world population (2 billion people) and causes 9 million new cases and about 2 million deaths each year worldwide (http://www.who.int/gtb/). The mechanisms by which *M. tuberculosis* causes disease have remained largely unknown until the improvement made recently *via* the application of modern molecular genetic tools, including genomic sequencing of the common lab virulent reference strain H37Rv [1], the clinical isolate CDC1551 [2], *M. bovis* [3] and *M. bovis* BCG [4] strains. However, comparative genomic analysis of paired virulent *M. tuberculosis* H37Rv strain *versus* the avirulent H37Ra strain has been lacking.

Historically, *M. tuberculosis* H37Ra is the avirulent counterpart of virulent strain H37Rv and both strains are derived from their virulent parent strain H37, which was originally isolated from a 19 year-old male patient with chronic pulmonary tuberculosis by Edward R. Baldwin in 1905 [5]. In order to obtain stable avirulent derivatives of H37, in 1935, William Steenken carried out a dissociation study based on aging of H37 bacilli on solid egg media [6]. The parental virulent H37 was inoculated onto solid egg media at pH 6.2. The resulting culture was allowed to age for 3–4 months at 37°C. By the end of the extended incubation, the original dry, discrete colonies lysed and transformed into a confluent viscous mass. In the midst of the viscous mass, secondary growth with different colony morphology emerged. The new growth, when picked and cultured on fresh media, produced no disease in guinea pigs [6], [7] and was designated H37Ra (“a” for avirulent). The virulent counterpart (*i.e*., H37 but with rough colony morphology) [5] was named H37Rv (“v” for virulent) and the original H37 was discontinued. Both H37Ra and H37Rv strains were maintained at Trudeau Institute, Saranac Lake, New York, for many years and were later deposited in the American Type Culture Collection (ATCC). H37Ra and its virulent counterpart H37Rv have been widely used as reference strains for studying virulence and pathogenesis of *M. tuberculosis* worldwide since 1940s and H37Ra is also used as an adjuvant to boost immunogenicity during immunization.

H37Ra has several characteristics that are different from its virulent sister strain H37Rv, including a raised colony morphology [6], loss of cord formation [8], loss of neutral red dye binding [9], decreased survival under anaerobic conditions [10], [11] or inside the macrophages [12], impaired ability to disrupt phagosomal membranes [13], and loss of virulence in guinea pigs [6], [7], [14] and mice [15], [16]. The distinguishing characteristics of H37Ra and H37Rv are maintained indefinitely on subculture, suggesting that the two strains differ genetically. Despite numerous biochemical and genetic studies in the past 70 years [9], [11], [17]–[26], the molecular basis for the attenuation of virulence in H37Ra has remained obscure.

In this study, we determined the whole genome sequence of the *M. tuberculosis* H37Ra strain from the American Type Culture Collection (ATCC25177). Comparative genomic analysis of H37Ra with its recently sequenced virulent counterpart H37Rv not only provides important insights into the basis of attenuation of virulence in H37Ra but also improves our understanding of virulence and pathogenesis of *M. tuberculosis*.

## Results

### Genomic Features of *M. tuberculosis* H37Ra and Its Global Comparison with the Pathogenic Counterpart H37Rv and CDC1551

**Figure 1:**
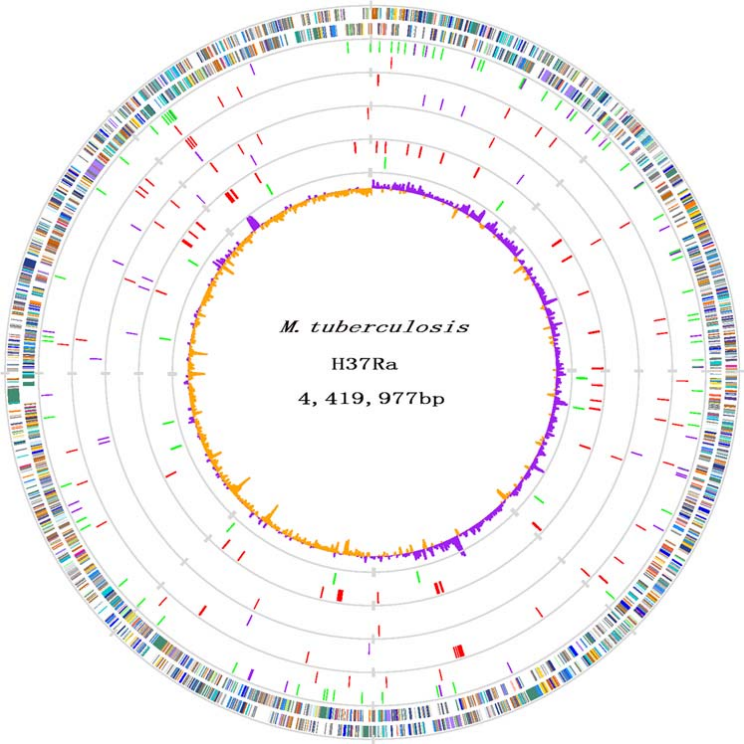
Atlas of the Chromosome of *M. tuberculosis* H37Ra ATCC25177 and its Comparison with *M. tuberculosis* H37Rv. Moving inside, each concentric circle represents genomic data for *M. tuberculosis* H37Ra and its comparison with *M. tuberculosis* H37Rv. The outer circle illustrates predicted coding sequences on the plus and minus strands, respectively, colored by functional categories according to COG classification. The 2^nd^ circle represents location of nucleotide substitution in coding regions (purple for synonymous substitutions; green for nonsynonymous substitutions) and noncoding regions (red), respectively. The 3^rd^ circle displays loci of insertions in H37Ra, distinguished by coding regions (red) and noncoding regions (purple). The 4^th^ circle shows loci of deletions in H37Ra, distinguished by coding regions (red) and noncoding regions (purple). The 5^th^ circle presents H37Ra specific genes compared with H37Rv and CDC151 genes, red for CDSs with variations and green for genes with promoter variations. The 6^th^ circle (innermost) represents GC skew (G−C)/(G+C) calculated using a 5 kb window.

*M. tuberculosis* H37Ra ATCC25177 contains a single circular chromosome of 4,419,977 bp with an average G+C content of 65.61% (GenBank accession number CP000611). A total of 4034 protein-coding sequences (CDS) are identified in the H37Ra genome with an average length of 1000 bp, representing 90.1% of the genome (Figure 1). Comparison with the H37Rv genome (GenBank accession AL123456) revealed a highly conserved gene content and order between these two strains. The base numbering start point of the H37Ra genome was chosen at the first base of the *dnaA* gene as in H37Rv, coinciding with the replication origin (*oriC*, Figure 1). The genome size of H37Ra is 8,445 bp larger than that of H37Rv (4,411,532 bp), as a result of 53 insertions and 21 deletions (indels, *i.e.*, insertions and deletions of any size) in H37Ra relative to H37Rv (see Table S1, S2 with notes). Besides these indels, 198 single nucleotide variations (SNVs) are identified between H37Ra and H37Rv (Table S3).

When all of the 272 genetic variations in H37Ra (including 198 SNVs, 53 insertions and 21 deletions) were compared with their corresponding loci in the clinical isolate CDC1551, it is interesting to note that the similarity between H37Ra and CDC1551 is higher than that between H37Ra and H37Rv (Table 1). Between H37Ra and CDC1551, 119 SNVs, 14 insertions and 5 deletions are the same, and thus, we designate these 138 variations as “H37Rv-specific”.

**Table 1:** Genetic variations between H37Ra and H37Rv and comparison with CDC1551

Genetic variations between H37Ra and H37Rv	Number of variations	Corresponding loci in CDC1551	
		Same as in H37Ra* (H37Rv-specific)	Same as in H37Rv (H37Ra-specific)
**Total Number**	272	138	88
**SNVs**	198	119	63
**Transitions**	102	69	26
**Transversions**	96	50	37
**Insertions (≥1bp)**	53	14	15
**Deletions (≥1bp)**	21	5	10

* Detailed information about variations that are the same between H37Ra and CDC1551 can be found in Tables S1, S2 and S3.

Since H37Ra and H37Rv both evolved from the same parental strain H37, it is unlikely that H37Ra could accumulate so many variations identical to CDC1551. Thus we re-sequenced the corresponding H37Rv genomic regions covering the 138 “H37Rv-specific” variation loci. Surprisingly, among the 85 successfully sequenced sites (including 72 SNV loci, 10 insertion loci and 3 deletion loci, while the unsuccessfully sequenced sites were mainly in the PE/PPE/PE-PGRS regions), only 3 SNV loci, 2 insertion loci and 1 deletion loci are the same as the published H37Rv genomic sequence, while the remaining 79 sites are identical to those of H37Ra and CDC1551 (Table S4).

The 6 real “H37Rv-specific” variations (*i.e*., those that are different from both H37Ra and CDC1551) are likely acquired during long-term repeated *in vitro* cultivation of H37Rv over the years but unrelated to the virulence property. Therefore, when all the 138 “H37Rv-specific” variations are excluded from the 272 genetic variations in H37Ra, we have only 134 total genetic variations that are “H37Ra-specific”, including 79 SNVs, 39 insertions and 16 deletions that are different from H37Rv or CDC1551. To ensure that these 134 “H37Ra-specific” variations are indeed unique to H37Ra and not due to possible sequencing errors of H37Rv sequence, we again re-sequenced these 134 “H37Ra-specific” variation loci using our strain of H37Rv (obtained from Trudeau Institute but maintained in mice, see Methods). We found 3 of the 79 SNVs and 1 of the 16 deletions to be the same as in H37Ra. Therefore, when these 4 sites together with all of the 138 “H37Rv-specific” variations are excluded from the total of 272 genetic variations that differ from H37Ra and H37Rv, only 130 genuine “H37Ra-specific” genetic variations, including 76 “H37Ra-specific” SNVs, 39 “H37Ra-specific” insertions and 15 “H37Ra-specific” deletions were further analyzed for their potential impacts on H37Ra phenotypes (Table S4).

Among the 76 SNVs, 2 are synonymous mutations, 8 are located in intergenic regions and predicted to have no effect on promoters. The remaining 66 SNVs might affect either promoter regions or CDSs of merely 32 genes, *i.e.*, 8 might affect the promoter of 12 genes (in some cases 1 SNV site is located in the probable promoter region of 2 divergently transcribed genes), and 58 affect the CDSs of 20 genes. Among the 39 “H37Ra-specific” insertions, 14 are located in the intergenic regions that might have no effect on gene promoters. For the remaining 25 insertions, 23 affect 19 CDSs while the other 2 might affect probable promoters of 2 genes. A total of 15 “H37Ra-specific” deletions might affect 12 genes, of which, 5 might affect probable gene promoters and 10 affect the CDSs. In sum, a total of 57 genes in H37Ra are likely affected by genetic variations and will be the focus of our functional analysis (Table 2).

**Table 2:** Complete list of “H37Ra-specific” genetic variations

H37Ra gene	H37Ra gene product	Variation	H37Rv Locus	H37Rv gene product
MRA_0011	IS6110 transposase	insertion	Rv0010c up	membrane protein
MRA_0012	IS6110 hypothetical protein	insertion	Rv0010c up	membrane protein
MRA_0040	conserved integral membrane protein	SNV	Rv0037c	integral membrane protein
MRA_0042 up	conserved transmembrane protein	SNV	Rv0039c up	transmembrane protein
MRA_0105	peptide synthetase	SNV	Rv0101	peptide syntherase Nrp
MRA_0131	PE-PGRS	SNV	Rv0124	PE-PGRS
MRA_0197	dihydroxy-acid dehydratase	SNV	Rv0189c	dihydroxy-acid dehydratase
MRA_0288	PE-PGRS family protein	insertion+deletion+SNV	Rv0279c	PE-PGRS
MRA_0391 up	conserved secreted protein	deletion	Rv0383c up	Conserved secreted protein
MRA_0585	PE-PGRS	SNV	Rv0578c	PE-PGRS
MRA_0646	hypothetical protein	deletion	Rv0635	hypothetical protein
MRA_0648	hypothetical protein	insertion	Rv0637	hypothetical protein
MRA_0767	two component system response transcriptional positive regulator PhoP	SNV	Rv0757	PhoP
MRA_0885	PPE family protein	insertion	Rv0878c	PPE
MRA_0887	marr-family transcriptional regulatory protein	SNV	Rv0880	Transcriptional regulatory protein(MarR)
MRA_0984	PE-PGRS	SNV	Rv0977	PE-PGRS
MRA_1014 up	para-aminobenzoate synthase component I	SNV	Rv1005c up	para-aminobenzoate synthase component I
MRA_1015 up	hypothetical protein	SNV	Rv1006 up	hypothetical protein
MRA_1029	hypothetical protein	SNV	Rv1021	hypothetical protein
MRA_1078	PE-PGRS family protein	SNV	Rv1068c	PE-PGRS
MRA_1102	PE-PGRS family protein	insertion+SNV	Rv1091	PE-PGRS
MRA_1106 up	PhoH-like protein PhoH2	SNV	Rv1095 up	PhoH2
MRA_1205A	PPE	deletion	Rv1196	PPE
MRA_1395 up	PE	SNV	Rv1386 up	PE
MRA_1459	PE-PGRS	insertion+SNV	Rv1450c	PE-PGRS
MRA_1766	putative phospholipase C 4 PlcD	insertion	Rv1755c up	PlcD
MRA_1767	IS6110 transposase	insertion	Rv1755c up	PlcD
MRA_1768	IS6110 hypothetical protein	insertion	Rv1755c up	PlcD
MRA_1768A	hypothetical protein	insertion	Rv1755c up	PlcD
MRA_1772	PE-PGRS	insertion	Rv1759c	PE-PGRS
MRA_1779	disrupted IS6110 transposase	insertion	Rv1765c	hypothetical protein
MRA_1815 up	PPE	SNV	Rv1802 up	PPE
MRA_1940	hypothetical protein	deletion	Rv1929c	hypothetical protein
MRA_2063	polyketide synthase Pks12	SNV	Rv2048c	polyketide synthase Pks12
MRA_2082 up	beta-lactamase	SNV	Rv2068c up	Beta-lactamase BlaC
MRA_2083 up	RNA polymerase sigma-70 factor	SNV	Rv2069 up	RNA polymerase sigma-70 factor
MRA_2113	PE-PGRS	insertion	intergenic	
MRA_2376	PPE	insertion	Rv2352c up	PPE
MRA_2420	PE-PGRS	SNV	Rv2396	PE-PGRS
MRA_2447 up	nicotinic acid mononucleotide adenyltransferase	SNV	Rv2421c up	nicotinic acid mononucleotide adenyltransferase
MRA_2678	IS6110 transposase	SNV	Rv2649	IS6110 transposase
MRA_2759 up	alanine and arginine rich protein	SNV	Rv2733c	alanine and arginine rich protein
MRA_2760 up	hypothetical protein	SNV	Rv2734	hypothetical protein
MRA_2849 up	hypothetical protein	insertion	Rv2825c up	hypothetical protein
MRA_3085 up	putative glutaredoxin NrdH	deletion	Rv3053c up	putative glutaredoxin NrdH
MRA_3225	hypothetical protein	deletion	intergenic	
MRA_3344 up	dihydrolipoamide dehydrogenase LpdA	insertion	Rv3303c up	dihydrolipoamide dehydrogenase
MRA_3384	PPE	deletion	Rv3343c	PPE
MRA_3391	PPE	insertion	Rv3350c	PPE
MRA_3428	PE-PGRS	deletion	Rv3388	PE-PGRS
MRA_3428A	hypothetical protein	insertion+SNV	Rv3389c	dehydrogenase
MRA_3547	PE-PGRS	SNV	Rv3507	PE-PGRS
MRA_3548	PE-PGRS	insertion	Rv3508	PE-PGRS
MRA_3553	PE-PGRS	insertion+deletion+SNV	Rv3514	PE-PGRS
MRA_3635	PE-PGRS	deletion	Rv3595c	PE-PGRS
MRA_3649 up	cell division protein FtsH	deletion+SNV	Rv3610c up	FtsH
MRA_3918	alanine and proline rich protein	SNV	Rv3879c	arginine and proline rich protein
Synonymous SNV in coding region				
MRA_2218	carbohydrate kinase CbhK	SNV	Rv2202c	carbohydrate kinase CbhK
MRA_3062	hypothetical protein	SNV	Rv3031	hypothetical protein

**Figure 2:**
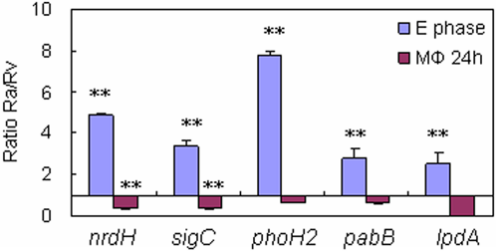
Transcription Analysis of Selected Genes with Promoter Mutations in H37Ra Compared to H37Rv in culture and in macrophages. The in vitro cultured *M. tuberculosis* H37Ra and H37Rv were grown to log phase. The bacterial cells in macrophages were harvested 24 hours after infection. Mycobacterial RNA was isolated and 16 S rRNA gene and *sigA* were used as internal controls for normalization of the transcriptional analysis for in vitro and macrophage derived mycobacteria, respectively. The transcription levels of the selected genes (*lpdA, pabB, phoH2, sigC* and *nrdH*) were determined by qRT-PCR as described in the Methods. Error bars were given as standard deviations (SDs) of the means. E phase: exponential (log) phase. Μφ: macrophage. The RT-PCR data were repeated at least three times. ** indicates p value <0.05.

To determine the impact of the sequence variations within the probable promoter regions of H37Ra, we performed real-time quantitative RT-PCR (qRT-PCR) to compare the levels of expression for several important candidate genes (*sigC*, *nrdH*, *phoH2*, *pabB*, *lpdA*) in H37Ra *vs* H37Rv both in culture and in macrophages. Interestingly, their expression levels were all increased *in vitro* but decreased in macrophages in H37Ra compared with H37Rv (p<0.05) (Figure 2).

### IS*6110* Related Genetic Variations between H37Ra and H37Rv and in Comparison to CDC1551

**Figure 3:**
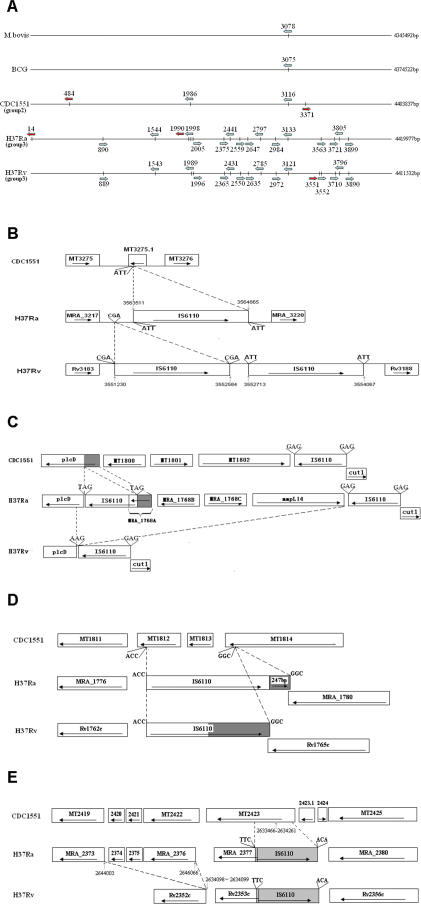
Schematic Diagram of IS*6110*-Mediated Genetic Variations Detected among H37Ra, H37Rv And CDC1551. A. IS*6110* distributions in the chromosomes of known *M. tuberculosis* complex strains. Blue arrow represents *IS6110* elements shared by more than one strain. Red arrow indicates IS*6110* elements specifically detected only in one strain. The direction of arrow indicates the transcription orientation of the transposase genes. The numbers above the arrows indicate the location (kb) of IS*6110* in the strains relative to the *oriC* in clockwise direction. B. Schematic diagram of a hot spot region for IS*6110* insertion. ‘ATT’, ‘CGA’ are the DRs flanking each IS*6110*, the same DRs on both sides of one IS*6110* indicate that no IS*6110* recombination had occurred here. In CDC1551, no IS*6110* is found in this region but instead, a gene MT3275.1 is present, which is disrupted by IS*6110* insertion(s) in H37Ra and H37Rv. C. Schematic diagram of the *plcD-cut1* region (RvD2) in H37Ra, H37Rv and CDC1551. The H37Ra CDSs are predicted to encode hypothetical proteins (MRA_1768A, and MRA_1768B), encoding a putative sulfite oxidase (MRA_1768C) and a putative transmembrane transport protein MmpL14 (MRA_1768D). ‘GAG’, ‘TAG’, ‘AAG’ are the DRs flanking the IS element. The coding sequences of MT1800, MT1801 and MT1802 of CDC1551 are identical to that of MRA_1768B, MRA_1768C and mmpL14 gene, respectively. D. Schematic diagram of the H37Ra MRA_1776-MRA_1780 region,with IS*6110* related 247 bp insertion (MRA_1779). Corresponding regions in H37Rv and CDC1551 are shown for comparison. The MT1812, MT1813 and 3′ end of MT1814 of CDC1551 correspond to the RvD3 region of H37Rv. The 247 bp remnant and its probable original counterpart in H37Rv are shaded. Referring to CDC1551 and the DRs, it might be inferred that two IS*6110* elements were once inserted in the RvD3 region in the process of evolving H37Ra and H37Rv. Subsequent recombination between these two IS elements caused deletion of RvD3 in H37Ra and H37Rv. The recombination in H37Ra underwent an incomplete exchange, leaving the remnant observed. E. Schematic diagram of the 2064 bp insertion region containing 2 *esat-6* like protein genes (MRA_2374, MRA_2375) and a PPE family protein gene (MRA_2376). A neighboring IS*6110* insertion is shown which might be H37-specific. H37Ra and H37Rv share the same IS*6110* insertion which disrupted the MT2423, generating a new gene, MRA_2377 (Rv2353c).

The H37Ra genome contain 32 complete and 10 disrupted IS elements (Table S5), almost identical to that of H37Rv except for slight differences in IS*6110*. In addition, only a limited number of Long Sequence Polymorphisms (LSPs, *i.e*., indels greater than 10 bp) [2] are identified between H37Ra and H37Rv and all of them are either IS*6110* related or in the PE/PPE/PE-PGRS family. There are 17 complete copies of IS*6110* in H37Ra, but 16 copies in H37Rv. The difference in IS*6110* copy number between H37Ra and H37Rv is due to two complete “H37Ra-specific” IS*6110* insertions, located at 14 kb [22] and 1990 kb away [27] from *oriC*, respectively (Figure 3A), in addition to one complete IS*6110* deletion (1358 bp, containing Rv3184 and Rv3185) in H37Ra located in a region which seems to be a hot spot for IS*6110* insertion, where H37Ra has only one but H37Rv has two copies of IS*6110* (Figure 3B). The absence of one copy of IS*6110* in H37Ra is unlikely to result from IS*6110* excision or IS*6110-*related recombination but rather due to acquisition of the IS*6110* in H37Rv, since the identical direct repeats (DRs) found flanking the IS*6110* at this location in H37Ra do not support the possibility of a recombination between two neighboring IS elements causing deletion of one copy of IS*6110* in H37Ra [28], [29].

There are five H37Rv related deletion (RvD) loci defined based on the genomic deletions identified in *M. tuberculosis* H37Rv compared to *M. bovis* and *M. bovis* BCG [27], and four of them are associated with IS*6110* [22]. Among these RvD loci, only the RvD2 of H37Ra is different from that of H37Rv, where an 8166 bp insertion is located between the 5′ proximal of *plcD* gene and the adjacent IS*6110* (Figure 3C). This fragment contains 6 complete CDSs with 2 of them (MRA_1767, MRA_1768) belonging to a complete IS*6110* located at 1990 kb from *oriC*. Further comparison with CDC1551 suggested that this fragment once existed in H37Rv, but was subsequently deleted after an IS*6110*-mediated recombination (Figure 3C). Assuming that CDC1551 retains more genomic characteristics of the common ancestor for H37Ra, H37Rv and CDC1551, an IS*6110* (1990795-1989441) is inserted between the 476^th^ and the 477^th^ bp of the *plcD* gene thus causing a 5′ truncation in the *plcD* gene of H37Ra (the 5′ part of *plcD* gene formed a new CDS, MRA_1768A). However, in H37Rv, the IS*6110* is inserted between the 696^th^ and the 697^th^ bp of the *plcD* gene and a subsequent homologous recombination between this IS*6110* and the adjacent IS*6110* located at the 5′ end of the *cut1* gene caused a deletion of an 8 kb fragment in H37Rv. This proposition is supported by analyzing the DRs flanking these IS*6110* elements. The ‘GAG’ adjacent to the right end of IS*6110* in H37Rv is similar to the DRs of IS*6110* next to the *cut1* gene in H37Ra and CDC1551, while the 3 bases adjacent to the left end of H37Rv IS*6110* is ‘AAG’, similar to the IS*6110* insertion site of *plcD* gene. Therefore, the two pieces of the once co-existing IS*6110* elements with DRs of ‘AAG’ and ‘GAG’ likely had undergone a recombination that generated one IS*6110* element carrying one DR of each parent in H37Rv.

Corresponding to the previously described RvD3 locus in H37Rv [22], [30], an IS*6110* remnant of 247 bp is found adjacent to this region in H37Ra, joining with a complete IS*6110* (Figure 3D). Referring to the corresponding region in CDC1551 and the sequence of DRs, one might assume that two IS*6110* elements were once inserted in this region of H37 and in the process of evolving, recombination between these two IS elements caused the deletion of RvD3 in H37Rv and H37Ra. Subsequent recombination in H37Ra underwent an incomplete exchange, leaving the 247 bp IS*6110* remnant as observed.

A 2064 bp insertion present in both H37Ra and CDC1551 but deleted from H37Rv is designated as RvD6 (Figure 3E). This fragment, which contains two genes (MRA_2374, MRA_2375) encoding *esat-6* like proteins and a gene encoding a PPE family protein (MRA_2376), is located at 1 bp upstream of the start codon of MRA_2373 (Rv2352c). The MRA_2376 is identical to MRA_2373, and the same gene duplication is observed in CDC1551 (MT2419 and MT2422). However, only one homologous gene (Rv2352c) is found in H37Rv. It is most likely that a recombination occurred between the two precursors of Rv2352c and induced the 2064 bp deletion in H37Rv.

### Mutations Affecting Transcription Factors and Global Regulations

The high degree of similarity in the genomic content and the apparently disparate phenotypes between H37Ra and H37Rv indicate that mutations in transcription factors and genes related to global regulations might account for its unique characteristic of growth and/or attenuated virulence of H37Ra. In fact, only limited mutations are found in this category.

A transversion (A-T) is identified in H37Ra, in the predicted promoter region, 51 bp upstream of the start codon of *sigC* (MRA_2083/Rv2069), which encodes the extracellular function (ECF) subfamily sigma factor [31] (Table S6). Our qRT-PCR result indicated that the transcription levels of *sigC* in H37Ra were up-regulated compared to H37Rv in log phase cultures, but the transcription level of *sigC* in H37Ra was nearly 4 times lower than H37Rv in macrophages (Figure 2). SigC is known to regulate the expression of at least 38 genes involved in a broad range of cellular processes and disruption of *sigC* resulted in a significant virulence attenuation in mice [31] and guinea pigs [32]. Therefore, decreased expression of *sigC* in H37Ra in macrophages might impact the global gene regulation related to its attenuation of virulence.

**Figure 4:**
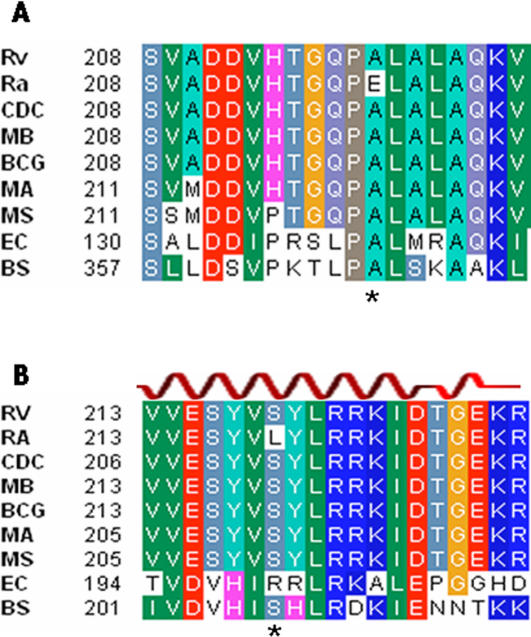
Multiple Sequence Alignment of MazG (A) and the C-Terminal Effector Domains of PhoP (B). The amino acid sequences used are from *M. tuberculosis* H37Rv (Rv), H37Ra (Ra), CDC1551(CDC), *M. bovis* (MB), *M. bovis* BCG (BCG), *M. avium* (MA), *M. smegmatis* (MS), *Escherichia coli* (EC) and *Bacillus subtilis* (BS). The numbers refer to the amino acid residues of MazG and PhoP. The key secondary structure element (α3-helix) of PhoP is indicated above the sequence. The site of substitution is indicated by an asterisk under the sequence.

Hyperphosphorylated guanine [(p)ppGpp], an important cellular second message controlling gene expression in stringent response, is required for long-term survival of *M. tuberculosis* under starvation and *in vivo* persistence [33], [34]. In *E. coli*, MazG regulates the MazEF-mediated stringent response by depletion of ppGpp [35]. Compared to the counterpart Rv1021 in H37Rv, the H37Ra MRA_1029 (MazG) has a missense mutation resulting in an A219E replacement located in the highly conserved-^218^PAL- motif of the MazG (Figure 4A). This motif is within the predicted H1 α-helix, conserved across the MazG enzyme family, and together with three other helices, they comprise the active site of triphosphate pyrophosphatases [36]. Significant charge alteration in this critical motif might affect the enzyme activity and in turn alter the stringent response.

The *phoP* (MRA_0767/Rv0757) gene is a well known virulence factor in *M. tuberculosis* [37] and encodes a transcription activator in response to the sensor kinase, PhoR, located at the cell surface. This two-component regulatory system controls the expression of virulence genes including genes involved in synthesis of cell wall lipids [38], [39]. The H37Ra PhoP has a single base substitution resulting in highly conserved Ser219 replaced by Leu, which is located in the DNA binding α3 helix region of PhoP (Figure 4B). This mutation could impair the DNA binding of and transcription activation by PhoP and may be partly related to attenuation of virulence in H37Ra.

### Mutations Affecting Genes Directly Related to Metabolism and *in vivo* Growth

Several variations, mostly in the probable promoter regions of genes related to primary metabolism (Table S6), might impair the proper transcription tested by the above qRT-PCR experiments and alter the capability of the bacilli to survive *in vitro* and/or *in vivo*. Both *pabB* (MRA_1014/Rv1005c) and *nrdH* (MRA_3085/Rv3053c) genes were poorly expressed in H37Ra at 24h post-infection inside macrophages, but their *in vitro* expression in culture was up-regulated (Figure 2). A T-A transversion at -66 bp of the translational start codon of *pabB* identified in H37Ra (Table S6) might affect this potential promoter region causing alteration of expression. The *pabB* gene encodes *para*-aminobenzoate synthetase component-I involved in the biosynthesis of *p*-aminobenzoate (PABA), a precursor of folate biosynthesis. Mutation in *pabB* was shown to cause attenuation of virulence in *Burkholderia pseudomallei* [40]. The *nrdH* gene encoding glutaredoxin had a 14 bp deletion in its potential promoter region compared to that of Rv3053c in H37Rv, where it forms a duplicated tandem repeat (Figure S1). The *nrdH* gene is likely located in an operon with *nrdI* (MRA_3084), *nrdE* (MRA_3083) and perhaps, with *nrdF2* (MRA_3080) as well. Due to the important role of the NrdEF2 complex in chromosome duplication and DNA repair [41], the alteration in expression of this *nrdHIEF2* operon might impact exit from dormancy or survival *in vivo*.

The *ilvD* (MRA_0197, Rv0189c) gene encoding dihydroxy-acid dehydratase, an essential enzyme for branch chain amino acid and pantothenate (coenzyme A) biosynthesis, has a highly conserved Val284 (GTA) being substituted by Gly284 (GGA) in H37Ra. Since this variation is not located in any specific domains and there is no branch chain amino acid related phenotype for H37Ra [42], the biological significance of this mutation remains to be determined.

In the intergenic region between *lpdA* (Rv3303c/MRA_3344) and Rv3304 (MRA_3345), a 58 bp tandem repeat unit is repeated twice in H37Rv and CDC1551, but three times in H37Ra (Figure S1). The additional repeat unit in H37Ra might influence the expression of *lpdA* and its downstream gene *glpD2* as we found a hairpin structure (**CACGCCGC** TGC**GCGGCGTG** ) within this sequence, which implies a potential binding site for regulatory proteins. Downstream of the *lpdA-glpD2* operon, there is a cluster of genes, *phoY1,* MRA_3341 (Rv3300c), *atsB* and *lpqC*, which might be affected by the 58 bp repeat if they were co-transcribed with the *lpdA-glpD2* operon. Within the potential promoter regions of *lpdA-glpD2* operon and the downstream *phoY1*, each has a SigF binding site, GGATTG-N_16_-GGGTAT for *phoY1* and GGTTC-N_16_-GGGTGC for *lpdA* (Figure S1). The *phoY1* encodes a probable transcriptional regulatory protein homologous to the PhoU protein involved in regulation of phosphate uptake and persister formation in *E. coli* [43]. Our qRT-PCR experiment indicated that the H37Ra *lpdA* transcript was hardly detected at 24 hr after macrophage infection, whereas in H37Rv, it was up-regulated significantly compared to *in vitro* control (Figure 2).

A G-A transition is identified in the probable promoter region of another gene involved in phosphate metabolism, *phoH2* (MRA_1106/Rv1095). This gene encodes a putative ATPase involved in phospholipid metabolism and RNA modification [44]. This promoter region mutation caused higher level of expression of *phoH2* in vitro but lower expression in macrophages in H37Ra than H37Rv (Figure 2).

FtsH is an ATP-dependent membrane bound Zn metalloprotease involved in degradation of short-lived regulatory proteins such as heat shock ^32^σ in the cytosol and unassembled proteins in the membrane [45]. A 106 bp deletion at −51 bp and a C to T transversion at −65 bp of the start codon of *ftsH* (MRA_3649/Rv3610c) might affect the expression of this gene in H37Ra.

### Mutations Altering Cell Envelope and Genes Encoding Secreted Proteins

Cell envelope proteins, cell wall associated polyketide lipids and secreted proteins are involved in virulence and host cell interaction and immune responses [38], [39], [46]. The genome of *M. tuberculosis* contains a significant number of genes (*pks*) devoted to polyketide synthesis [1]. Two of them are mutated in H37Ra compared to H37Rv.

The *pks12* (MRA_2063/Rv2048c) is involved in dimycocerosyl phthiocerol synthesis and the disruption of this gene in H37Rv caused high degree of attenuation of virulence in mice [47]. Missense mutations T**C**A-T**T**A and **C**CA-**G**CA in MRA_2063 compared to Rv2048c cause S3004L and P1056A substitutions, respectively. The latter is in the conserved motif -^1055^WPP- within the linker peptide connecting two domains of acyl transferase and dehydratase, and is very likely to be deleterious.

The *nrp* (MRA_0105/Rv0101) gene encodes a non-ribosomal peptide synthetase involved in polyketide synthesis [48]. The H37Ra *nrp* gene has a nonsense mutation at its N-terminus, where the ^93^Trp codon TGG is replaced by a stop codon TAG(*), causing a large truncation of 2420 amino acids at the C-terminus, which is expected to disrupt the protein function and affect the synthesis of PDIM.

In H37Ra, the MRA_0040 has a G177D mutation in the putative transmembrane region leading to loss of two transmembrane-helices in Rv0037 of H37Rv. Sequence analysis revealed that this protein belongs to the major facilitator superfamily (MFS) involved in transporting small solutes in response to chemiosmotic ion gradients. Furthermore, a 55 bp deletion is identified at the −66 bp from the start codon of H37Ra MRA_0391 relative to H37Rv Rv0383c (Table S6). Although it probably encodes a conserved secreted protein, its biological function is unknown.

### Alterations in the PE/PPE/PE-PGRS Family Proteins

We found 35 PE/PPE/PE-PGRS family members that differ between H37Ra and H37Rv resulting from SNVs and indels (Table S7). MRA_1772 has a 1 bp insertion which induced a premature termination relative to its H37Rv ortholog Rv1759c encoding a fibronectin binding PE-PGRS family protein [49], causing it to be 2398 bp shorter than the original stop codon. MRA_3407 has an in-frame insertion of 3 amino acids at the C-terminus relative to its ortholog Rv3367 encoding the PE_PGRS51 protein in H37Rv. These H37Rv ortholog proteins are recognized by antibodies from TB patients [50].

MRA_0754 has five amino acid alterations in the PGRS domain compared to its ortholog Rv0746, which was induced during infection [51]. MRA_1205a has a 1 bp deletion at the 922^th^ base, causing frameshift and premature termination at the C-terminus in H37Ra relative to its ortholog Rv1196 (PPE18), which has previously been shown to induce gamma interferon production in infected or BCG-vaccinated calves [52]. MRA_3384, encoding a PPE family protein, lacks 576 amino acids in the C terminus compared to its ortholog Rv3343c due to a 1728 bp deletion.

Several PE/PPE/PE-PGRS genes seem to be preferential “hot spots” for mutations, including MRA_0288 (Rv0279), MRA_0395 (Rv0388c), MRA_0754 (Rv0746), MRA_3548 (Rv3508), MRA_3553 (Rv3514) (Table S7). MRA_3553 is a highly variable gene and has 25 SNVs (altering 21 amino acids), 2 deletions of 240 bp and 603 bp and one insertion of 9 bp relative to its ortholog Rv3514. MRA_3548 is 1068 bp shorter than its ortholog Rv3508 due to a 402 bp insertion causing premature termination and 5 SNVs and 7 insertions. Notably, these two genes show extensive variations when compared to strain CDC1551 and other *M. tuberculosis* strains.

## Discussion

Despite numerous studies in the past several decades aimed at elucidating the mechanisms of virulence attenuation in H37Ra, the genetic basis for the attenuation has remained largely unknown. In this study, we determined the whole genome sequence of the attenuated *M. tuberculosis* strain H37Ra, performed comparative genomic analysis of H37Ra against its virulent counterpart H37Rv, and studied their genetic variations in reference to the clinical isolate CDC1551 genome sequence. These analyses provide accurate genomic information regarding the genetic differences between H37Ra and H37Rv, which are useful for better understanding of the pathogenesis of *M. tuberculosis* and basis for virulence attenuation in H37Ra.

**Figure 5:**
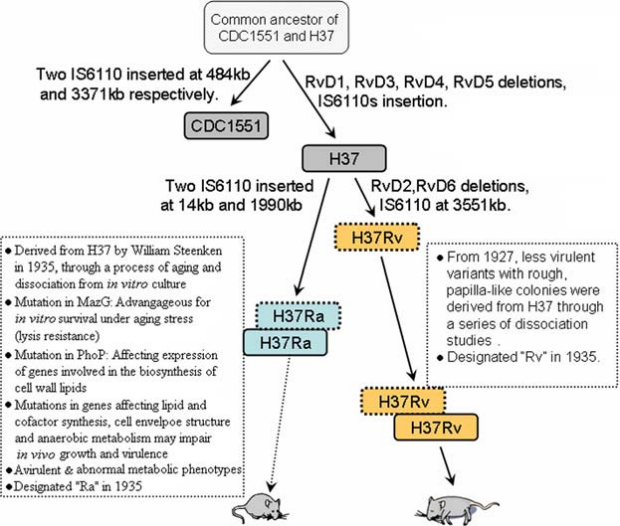
A Proposed Phylogenetic Relationship of H37Ra, H37Rv, H37, and the Closely Related Clinical Isolate CDC1551. The process of strain evolution as well as major phenotypes and related genotypes are illustrated in the text. The origination of H37Rv and H37Ra is described according to the literature [69]. Multiple appearances of both H37Ra and H37Rv represent their possible repeated *in vitro* passage, which may cause subsequent genetic variations.

Our analysis indicates that IS*6110* related indels and associated LSPs (*e.g.*, RvD6) among H37Ra, H37Rv and CDC1551, may serve as effective molecular markers to trace their phylogenetic relationship (Figure 5). These markers not only confirmed that CDC1551 is closely related to H37, the direct ancestor of H37Rv and H37Ra, but also indicated that H37Ra might retain more characteristic genetic features of H37 than H37Rv. The H37Rv specific RvD2 and RvD6 most probably occurred after H37Ra and H37Rv diverged from H37, while RvD1, RvD3, RvD4 and RvD5 may have occurred before H37 was evolved or H37 and CDC1551 diverged.

To exclude the mutations accumulated after the original isolation of H37Ra that are not involved in virulence attenuation, we freshly ordered the ATCC H37Ra strain ATCC21577 specifically for the genome sequencing in this study. H37Ra ATCC21577 was deposited in 1960s [16] and has not undergone significant genetic variations due to lack of subculture. The use of ATCC21577 H37Ra also minimizes undesired genetic changes unrelated to its original mutations involved in virulence attenuation, which is endorsed by the finding that only 272 genetic variations were identified between H37Ra and H37Rv, a relatively small number of sequence differences compared to 736 SNVs identified between the attenuated strain BCG Pasteur and *M. bovis* AF2122/97 [4]. Through referring the variations to strain CDC1551 and re-sequencing efforts, we further excluded 138 H37Rv-specific variations that are unlikely to be important for virulence and focused our analysis on only 66 SNVs, 25 insertions and 15 deletions that are specific between H37Ra and H37Rv and may affect CDSs or putative promoters (Table 2). It is surprising that H37Rv has so many (138 out of 272) SNVs that are different from H37Ra and CDC1551. Several explanations are possible. First, the H37Rv strain used for the primary sequencing [1] had probably accumulated various mutations during repeated *in vitro* passages. Second, sequence assembly from different H37Rv libraries (cosmid and plasmid libraries) constructed at different times over several years from different batches of H37Rv in the original H37Rv sequencing project [1] may also be a source of discrepancy. In addition, possible sequencing errors in the original H37Rv genome sequence may also be involved.

Through a process of aging for 3–4 months, William Steenken caused the dissociation of avirulent strain from H37 and other fresh clinical isolates [6], [7]. The multiple phenotypic alterations of H37Ra [6], [8]–[13] and the failure to fully restore the virulence of H37Ra by complementation with a cosmid library of H37Rv [18] suggest that H37Ra must have multiple mutations underlying its attenuation of virulence. Unlike BCG where multiple large chromosomal deletions underlie its virulence attenuation [19], [55], our whole genome sequencing analysis indicated that H37Ra has no gross deletions but instead has about 8 kb more DNA than H37Rv. This finding explains why the previous genome subtractive hybridization that led to identification of large deletions in BCG has failed to identify any deletion in H37Ra compared with H37Rv [19].

Our comparative genomic studies provided, for the first time, a molecular roadmap that allows us to address the basis of virulence attenuation and the multiple phenotypic changes of H37Ra. Although multiple mutations in noncontiguous chromosomal loci of H37Ra collectively lead to multiple phenotypic changes including virulence attenuation, it is likely that one or two initial mutations affecting global metabolic regulation might provide some advantage for *in vitro* survival under the conditions of aging and cell lysis (Figure 5). The missense mutations affecting MazG and/or PhoP in H37Ra are likely the candidates for the proposed primary alterations.

Although the impact of the *mazG* missense mutation (A219E) in H37Ra is yet to be determined, it may be inferred from the regulatory role of MazG upon the MazEF mediated bacteriostasis *via* depletion of ppGpp as established in *E. coli* [35]. There are at least 7 *mazF* genes in *M. tuberculosis*, 4 of which caused cell growth arrest when expressed in *E. coli* [54]. If the only MazG in *M. tuberculosis* has a similar function as in *E. coli* and the A219E mutation of H37Ra MazG does alter its function, the cellular ppGpp level in H37Ra and the MazF (or other toxin/antitoxin systems) mediated persistence might be altered as well. Therefore, the MazG mutation might be critical for the original emergence of H37Ra after aging. Further studies are needed to determine if this mutated MazG provides an advantage for H37Ra to survive during the aging-mediated cell lysis and to allow subsequent accumulation of multiple mutations for better *in vitro* growth.

It has been reported that H37Ra lost the ability to synthesize sulpholipids (SL), polyacyltrehalose (PAT) and phthiodiolone dimycocerosate ester (PDIM) [26], [56]. In accordance with the known changes in cell wall lipids of H37Ra, we identified several genes involved in cell wall lipid synthesis that are mutated in H37Ra. PhoP is a positive transcriptional regulator that controls the expression of many genes including *pks2* and the *msl3* gene clusters involved in the biosynthesis of complex cell wall lipids such as SL, PAT and diacyltrehalose [38], [39]. Inactivation of PhoP caused attenuated virulence in *M. tuberculosis* [37], loss of cell wall lipids and cording property, and neutral red binding ability [38], [39]. We identified in H37Ra an S219L mutation in the DNA binding α3 helix region of the PhoP protein. A recent study has shown that although the PhoP S219L mutation is responsible for loss of synthesis of some cell wall lipids and altered morphology of H37Ra, it did not account for the loss of neutral red binding, the production of virulence lipid PDIM and attenuated virulence since complementation of H37Ra with *phoP* has failed to confer these phenotypes [56].

Our study found other mutations besides PhoP that could be responsible for the loss of virulence in H37Ra. The PDIM synthesis defect in H37Ra and the loss of neutral red binding could be due to a severe truncation in the *nrp* gene (non-ribosomal peptide synthetase) and the two mutations in *pks12* identified in this study (Table 2, Table S3), which are expected to affect the virulence of H37Ra. In *M. bovis,* the *nrp* (Mb0104) is the last gene of an operon Mb0099-Mb0104 (*ppe1-nrp*) involved in the synthesis of phthiocerol and PDIM [48], [57]. Transposon insertion into Mb0100 in *M. bovis* caused a polar effect on the expression of the downstream genes including Mb0104, which led to altered morphology and attenuation of virulence in guinea pigs [48], suggesting *nrp* is an important virulence factor.

It is well known since 1950s that avirulent strain H37Ra has a defect in anaerobic metabolism and survives much less well than virulent H37Rv *in vivo* or under anaerobic conditions [10], [11]. The molecular basis for this phenomenon is unknown. We have shown that H37Ra has a 58 bp repeat DNA insertion in the putative promoter region of the *lpdA-glpD2* operon, which could affect the expression of both LpdA and GlpD2. The differential expression phenotype of *lpdA* transcription revealed by our qRT-PCR experiment indicated that the *lpdA* was probably an early response gene involved in adaption to macrophage environment. The *M. tuberculosis* LpdA (Rv3303c), despite its significant homology to other LpdA enzymes, does not have lipoamide dehydrogenase activity but instead has transhydrogenase activity and quinone reductase activity that enables transfer of reducing power from the reduced pyridine nucleotide pool to the electron transport chain [58], which could be important for energy production under anaerobic conditions. LpdA is a virulence factor involved in removing reactive oxygen species released by the host cells and thus contributing to *in vivo* virulence [59]. Although GlpD2 (Rv3302c) is annotated as glycerol-3-phosphate dehydrogenase for aerobic respiration, its location in an operon with anaerobic respiration enzyme LpdA suggests that GlpD2 may be functionally more akin to anaerobic glycerol-3-phosphate dehydrogenase (GlpA) involved in anaerobic respiration. The 58 bp repeat DNA insertion in the putative promoter region of the *lpdA-glpD2* could affect the expression of *lpdA* and *glpD2* and may cause reduced survival of H37Ra under reduced oxygen tension in host tissues.

Apart from *lpdA*, other genes with promoter region mutations may also be involved in virulence attenuation since those genes in H37Ra had different expression patterns compared to H37Rv (Figure 2). Since SigC is a virulence factor in *M. tuberculosis* [31], [32], its altered expression caused by a promoter mutation may impact its global regulatory effect and may be associated with virulence attenuation in H37Ra. In addition, *M. tuberculosis* NrdEF2 complex forms the class Ib oxygen-dependent ribonucleotide reductase, which catalyzes the reduction of ribonucleotides to deoxyribonucleotides and is considered essential due to the failure of obtaining null mutants in *M. tuberculosis* [41]. In this study, the expression of the *nrdHIEF2* operon was shown to be altered in H37Ra compared to that in H37Rv (Figure 2). It is thus significant to correlate this mutation with survival *in vivo* with respect to the possible role of NrdEF2 in DNA repair, which is expected to occur during exit from dormancy.

**Figure 6:**
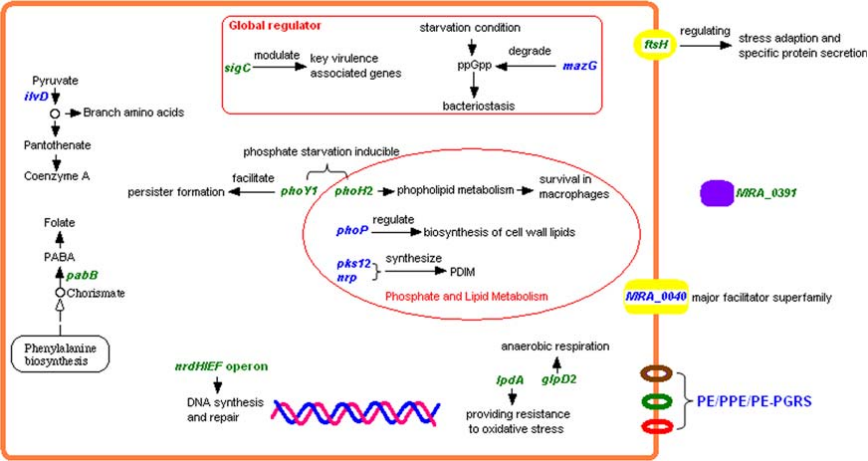
Schematic Diagram of Major H37Ra Genes with Functions Affected by Mutations. The genes in blue and in green indicate variations that occurred in coding sequences and in promoter regions, respectively.

In summary, through successful combination of sequencing genomics, comparative genomics and functional genomics strategies, our study has provided, for the first time, a comprehensive description of possible genetic variations, likely multiple mutations in H37Ra, which may account for its attenuation of virulence and various other phenotypic changes that are different from its virulent counterpart H37Rv. Besides variations in the PE/PPE/PE-PGRS genes, which have been shown to encode proteins with diverse functions such as virulence, fibronectin binding, and cell surface antigenic variations to evade the immune system [49], [60], [61], missense/nonsense mutations in genes coding for proteins related to stress response (*mazG*), transcription activation of lipid biosynthesis (*phoP*), amino acid biosynthesis (*ilvD*), polyketide synthesis (*pks12* and *nrp*) may also contribute to loss of virulence in H37Ra. Our qRT-PCR experiments validated that most genes with promoter mutations in H37Ra were up-regulated *in vitro* but down-regulated in macrophages relative to H37Rv. These promoter mutations may affect transcription of operons encoding genes related to energy metabolism (*lpdA-glpD2*), cofactor biosynthesis (*pabB*), nucleotide metabolism (*nrdH*/*nrdE*/*nrdF2*), lipid metabolism (*phoH2*), protein degradation (*ftsH*), and sigma factors (*sigC*), which may all contribute to the loss of virulence in H37Ra (Figure 6). Further biochemical, molecular genetic and in vivo studies are required to confirm and assess the relative importance of these mutations in causing the attenuation of virulence in H37Ra. Such studies will not only improve our understanding of mechanisms of pathogenesis of *M. tuberculosis* but also facilitate design of new vaccines and new therapeutic agents based on the identified virulence-associated mutations.

## Materials and Methods

### Bacterial strain and genome sequencing

*M. tuberculosis* strain H37Ra (ATCC25177) was freshly obtained from American Type Culture specifically for the purpose of genome sequencing in this study. The H37Ra was grown in Middlebrook 7H9 liquid medium with ADC (albumin-dextrose-catalase) supplement to late log phase for preparation of high-molecular weight genomic DNA as described [62]. Shotgun sequencing approach was performed employing three independent genomic libraries with average DNA fragments of 2–3 kb, 6–8 kb and 8–10 kb, respectively, constructed in the pSMART-LC vector (Lucigen Corporation). A total of 44,366 reads, or about 7 times coverage of the genome, were separately generated from the libraries by sequencing both ends of the clones. Arachne [63] and Phrap (www.phrap.org) were used for sequence assembly. Sequence gaps were filled in by first determining the order of supercontigs using multiplex PCR [64] and then sequencing the PCR products *via* primer walking or shotgun sequencing. The H37Rv strain used in this study for resequencing to resolve the discrepancies in the published H37Rv genome sequence [1] was obtained from Johns Hopkins for TB Research (E. Nuermberger, J. Grosset, W. Bishai, who obtained this strain in 1998 from Frank Collins then at Trudeau Institute where H37Rv was originally isolated by William Skeenken in 1935). This H37Rv strain has been maintained in mice to prevent loss of virulence and is not subjected to repeated in vitro cultivation.

### Sequence annotation and bioinformatic analysis

CDSs were identified by ZCURVE 1.0 [65]. Transfer RNA genes were predicted by tRNAscan-SE [66]. Functional annotation of CDSs was performed through comparisons to public and in-house databases using BLASTP, followed by manual curation. Comparative genome analysis was performed by using the Artemis Comparison Tool (ACT; www.sanger.ac.uk_Software). Promoter region was analyzed by Neural Network Promoter Prediction (http://www.fruitfly.org/seq_tools/ promoter.html). The atlas of genome is drawn using GenomeViz1.1 [67].

### Quantitative RT-PCR

*M. tuberculosis* H37Rv and H37Ra were grown in Middlebrook 7H9 broth with albumin-dextrose-catalase supplement (Becton Dickinson) to log phase. The THP-1 macrophage cell culture was performed as previously described [68]. THP-1 cells were infected with exponential bacterial suspensions of H37Rv and H37Ra at multiplicity of infection of 10:1. After incubating the mixture for 1.5 h at 37°C extracellular bacteria and non-adhered cells were removed by washing with RPMI medium. RNA was extracted from H37Rv and H37Ra in vitro or post macrophage infection. Random primers were used to synthesize first strand cDNA using the SuperScript II RT system (Invitrogen). Real-time quantitative PCR reactions were performed using the SYBR Green PCR Kit (TOYOBO) following the manufacturer's protocol. The reactions were carried out in a 96-well reaction plate and reporter fluorescence was measured on an iCycler real-time PCR system (Bio-Rad) using the following thermal cycler conditions: denaturation 1 cycle, 95°C for 2 min; PCR amplification, 40 cycles, 95°C for 30 s, 65°C for 20 s, 72°C for 30 s; final extension, 1 cycle, 72°C for 5 min. Relative transcript levels of the target genes were calculated by normalizing the levels of RNA of target genes with the level of 16 S rRNA gene or *sigA* expression in the same sample. Changes in target gene's mRNA levels between H37Ra and H37Rv were illustrated by the ratio of relative transcription levels of H37Ra compared to H37Rv.

### Statistical analysis

The RT-PCR data of the transcription levels of selected genes with promoter mutations were subjected to statistical analysis. Significance was defined according to p-values calculated from Student *t*-test analysis of ΔCt values of the selected genes between H37Ra and H37Rv. p-value<0.05 was considered to have statistical significance.

## Supporting Information

Figure S1 (0.19 MB DOC)

Table S1 (0.18 MB DOC)

Table S2 (0.09 MB DOC)

Table S3 (0.60 MB DOC)

Table S4 (0.34 MB DOC)

Table S5 (0.05 MB DOC)

Table S6 (0.08 MB DOC)

Table S7 (0.07 MB DOC)
