# Supplementary figures and images for: Genetic Basis of Virulence Attenuation Revealed by Comparative Genomic Analysis of Mycobacterium tuberculosis Strain H37Ra versus H37Rv
Source: PLoS One. 2008 Jun 11;3(6):e2375. doi: 10.1371/journal.pone.0002375 (PMC2440308; doi:10.1371/journal.pone.0002375)

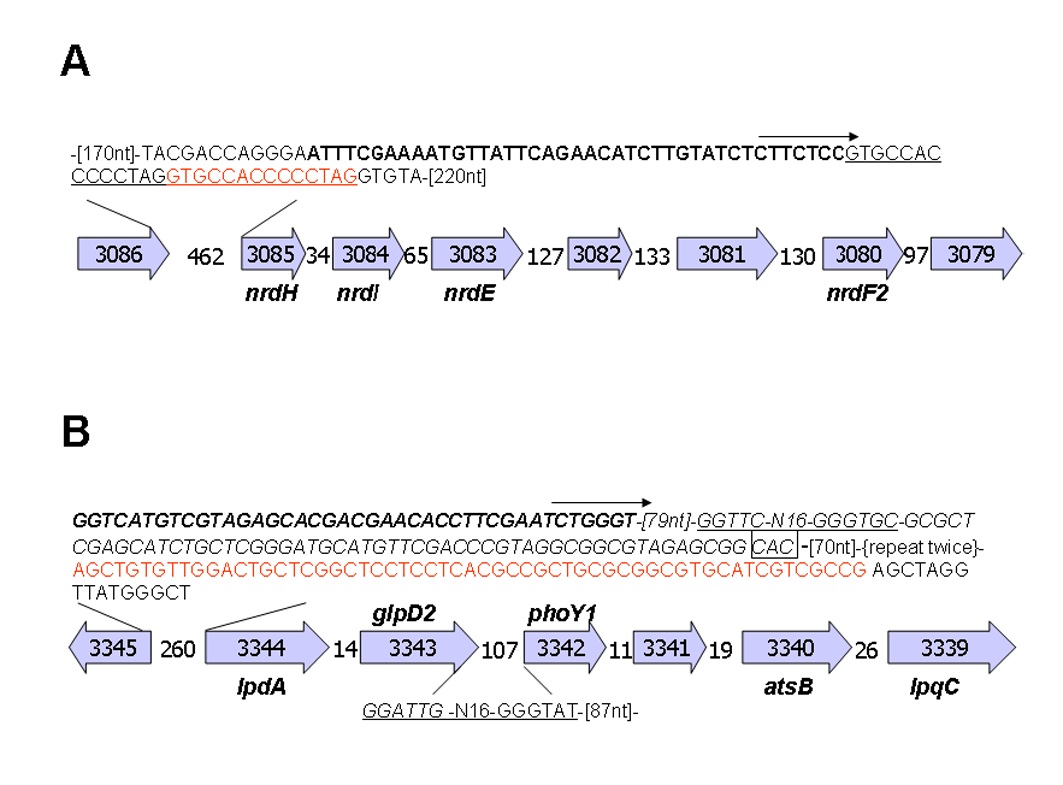

Supplement: Figure S1 — (0.19 MB DOC) [file pone.0002375.s001.tif]
