# Supplementary material for: Genetic Basis of Virulence Attenuation Revealed by Comparative Genomic Analysis of Mycobacterium tuberculosis Strain H37Ra versus H37Rv
Source: PLoS One. 2008 Jun 11;3(6):e2375. doi: 10.1371/journal.pone.0002375 (PMC2440308; doi:10.1371/journal.pone.0002375)
Supplement: Table S1 — (0.18 MB DOC) [file pone.0002375.s002.doc]

**Table S1. Insertions detected in H37Ra compared to H37Rv**

|  | **Insertion in H37Ra** | | | **Insertions same  as in CDC1551** | **Insertion site in H37Rv** | | |
| --- | --- | --- | --- | --- | --- | --- | --- |
|  | **Coordinates (length)** | **Locus** | **Gene name or product** |  | **Coordinates** | **Locus** | **Gene name or product** |
| **Ⅰ** | 132535 (1bp) |  |  | yes | 131176-131177 |  |  |
|  | 178886 (1bp) |  |  |  | 177526-177527 |  |  |
|  | 1314649 (1bp) |  |  | yes | 1313338-1313339 |  |  |
|  | 2217491 (1bp) |  |  | yes | 2207591-2207592 |  |  |
|  | 2533110-2533112 (3bp) |  |  |  | 2523207-2523208 |  |  |
|  | 3135094-3135095 (2bp) |  |  |  | 3123125-3123126 |  |  |
|  | 3135101 (1bp) |  |  |  | 3123130-3123131 |  |  |
|  | 3135111 (1bp) |  |  |  | 3123139-3123140 |  |  |
|  | 3135118-3135119 (2bp) |  |  |  | 3123145-3123146 |  |  |
|  | 3135125-3135126 (2bp) |  |  |  | 3123150-3123151 |  |  |
|  | 3135135 (1bp) |  |  |  | 3123158-3123159 |  |  |
|  | 3135140-3135141 (2bp) |  |  |  | 3123162-3123163 |  |  |
|  | 3135152-3135154 (3bp) |  |  |  | 3123172-3123173 |  |  |
|  | 3135159-3135160 (2bp) |  |  |  | 3123176-3123177 |  |  |
|  | 3135164 (1bp) |  |  |  | 3123179-3123180 |  |  |
|  | 3135174-3135179 (6bp) |  |  |  | 3123188-3123189 |  |  |
|  | 3145568 (1bp) | MRA_2849  upstream | hypothetical protein |  | 3133576-3133577 | Rv2825c  upstream | hypothetical protein Rv2825c |
|  | 3348673-3348849 (177bp) |  |  |  | 3336680-3336681 |  |  |
|  | 3601469 (1bp) |  |  | yes | 3590686-3590687 |  |  |
|  | 3701737-3701794 (58bp) | MRA_3344  upstream | dihydrolipoamide dehydrogenase LpdA |  | 3690953-3690954 | Rv3303c  upstream | dihydrolipoamide dehydrogenase |
| **Ⅱ** | 13620-14977 (1358bp) | MRA_0011 | IS6110 transposase |  | 13619-13620 |  |  |
|  |  | MRA_0012 | IS6110 hypothetical protein |  |  |  |  |
|  | 1989219-1997384 (8166bp) | MRA_1766 | putative phospholipase C 4 PlcD |  | 1987700-1987701 |  |  |
|  |  | MRA_1767 | IS6110 transposase |  |  |  |  |
|  |  | MRA_1768 | IS6110 hypothetical protein |  |  |  |  |
|  |  | MRA_1768A | hypothetical protein |  |  |  |  |
|  |  | MRA_1768B | conserved hypothetical protein |  |  |  |  |
|  |  | MRA_1768C | putative sulfite oxidase |  |  |  |  |
|  |  | MRA_1768D | putative transmembrane transport protein MmpL14 |  |  |  |  |
|  | 2644003-2646066 (2064bp) | MRA_2374 | putative esat-6 like protein |  | 2634098-2634099 |  |  |
|  |  | MRA_2375 | putative esat-6 like protein |  |  |  |  |
|  |  | MRA_2376 | PPE family protein |  |  |  |  |
| **Ⅲ** | 235857-235858 (2bp) |  |  | yes | 234496-234497 | Rv0197 | POSSIBLE OXIDOREDUCTASE |
|  | 3944494-3944596 (103bp) |  |  |  | 3935414-3935415 | Rv3508 | PE-PGRS FAMILY PROTEIN |
|  | 3944602-3944623 (22bp) |  |  |  | 3935419-3935420 |  |  |
|  | 3944628-3944665 (38bp) |  |  |  | 3935423-3935424 |  |  |
|  | 3944672-3944678 (7bp) |  |  |  | 3935429-3935430 |  |  |
|  | 3944686-3944762 (77bp) |  |  |  | 3935436-3935437 |  |  |
|  | 3944773-3944814 (42bp) |  |  |  | 3935446-3935447 |  |  |
|  | 3944828-3944950 (123bp) |  |  |  | 3935459-3935460 |  |  |
| **Ⅳ** | 2007141-2007387 (247bp) | MRA_1779 | disrupted IS6110 transposase |  | 1997455-1997456 | Rv1765c | hypothetical protein Rv1765c |
|  | 338056 (1bp) | MRA_0288 | PE-PGRS family protein |  | 336694-336695 | Rv0279c | PE-PGRS FAMILY PROTEIN |
|  | 1634841-1635047 (207bp) | MRA_1459 | PE-PGRS family protein |  | 1633530-1633531 | Rv1450c | PE-PGRS FAMILY PROTEIN |
|  | 3788786-3788794 (9bp) | MRA_3407 | PE-PGRS family protein | yes | 3779671-3779672 | Rv3367 | PE-PGRS FAMILY PROTEIN |
|  | 3951008-3951016 (9bp) | MRA_3551 | PE-PGRS family protein | yes | 3941516-3941517 | Rv3511 | PE-PGRS FAMILY PROTEIN |
|  | 3957589-3957597 (9bp) | MRA_3553 | PE-PGRS family protein |  | 3948328-3948329 | Rv3514 | PE-PGRS FAMILY PROTEIN |
|  | 734218 (1bp) | MRA_0648 | hypothetical protein |  | 732912-732913 | Rv0637 | hypothetical protein Rv0637 |
|  | 1011514 (1bp) | MRA_0914 | penicillin-binding protein 4 | yes | 1010206-1010207 | Rv0907 | hypothetical protein Rv0907 |
|  | 2002016 (1bp) | MRA_1772 | PE-PGRS family protein |  | 1992331-1992332 | Rv1759c | PE-PGRS FAMILY PROTEIN |
|  | 2366894 (1bp) | MRA_2113 | PE-PGRS family protein |  | 2356993-2356994 | Rv2098c | PE-PGRS FAMILY PROTEIN (frameshifted) |
|  | 2366909 (1bp) |  |  |  | 2357007-2357008 |  |  |
|  | 3392609 (1bp) | MRA_3052 | PPE family protein | yes | 3380439-3380440 | Rv3021c | PPE FAMILY PROTEIN |
|  | 3776134 (1bp) | MRA_3391 | PPE family protein |  | 3767020-3767021 | Rv3350c | PPE FAMILY PROTEIN |
|  | 425685 (1bp) | MRA_0363 | PPE family protein | yes | 424322-424323 | Rv0354c | PPE FAMILY PROTEIN |
|  | 468809 (1bp) | MRA_0395 | PPE family protein | yes | 467500-467501 | Rv0388c | PPE FAMILY PROTEIN |
|  | 468819 (1bp) |  |  | yes | 467509-467510 |  |  |
|  | 1170026 (1bp) | MRA_1055 | hypothetical protein | yes | 1168717-1168718 | Rv1046c | hypothetical protein Rv1046c |
|  | 1782104 (1bp) | MRA_1586 | putative phiRv1 phage protein | yes | 1780586-1780587 | Rv1575 | Probable phiRV1 phage protein |
|  | 978213 (1bp) | MRA_0885 | PPE family protein |  | 976906-976907 | Rv0878c | PPE FAMILY PROTEIN |
|  | 1219277 (1bp) | MRA_1102 | PE-PGRS family protein |  | 1217967-1217968 | Rv1091 | PE-PGRS FAMILY PROTEIN |
|  | 3812636-3812637 (2bp) | MRA_3428A | hypothetical protein |  | 3803959-3803960 | Rv3389c | POSSIBLE DEHYDROGENASE |
|  | 3944088-3944489 (402bp) | MRA_3548 | PE-PGRS family protein |  | 3935410-3935411 | Rv3508 | PE-PGRS FAMILY PROTEIN |

Note: Twenty insertions are located in the intergenic regions, with two possibly affecting the promoter of genes (Part I of Table S1). The length of these insertions ranges from 1 to 6 bp in most cases except for one 58 bp and one 177 bp insertions. Three insertions located in the intergenic regions of H37Rv introduced 11 complete ORFs and an extension to the 5’ end of the *plcD* gene (Rv1755c) in H37Ra (MRA_1766, Part II of Table S1). Eight insertions are within genes in H37Rv which lead to gene disruptions in H37Ra (Part III of Table S1). The remaining 22 insertions (Part IV of Table S1) are located in 20 H37Rv genes and alter the orthologous ORFs in H37Ra, with 14 of them belonging to the PE/PPE/PGRS family.
