# Supplementary material for: Genetic Basis of Virulence Attenuation Revealed by Comparative Genomic Analysis of Mycobacterium tuberculosis Strain H37Ra versus H37Rv
Source: PLoS One. 2008 Jun 11;3(6):e2375. doi: 10.1371/journal.pone.0002375 (PMC2440308; doi:10.1371/journal.pone.0002375)
Supplement: Table S2 — (0.09 MB DOC) [file pone.0002375.s003.doc]

**Table S2. Deletions detected in H37Ra compared to H37Rv.**

|  | **Deletion site in H37Ra** | | | **Deletion same  as in CDC1551** | **Deletion in H37Rv** | | |
| --- | --- | --- | --- | --- | --- | --- | --- |
|  | **Coordinates (length)** | **Locus** | **Gene name or product** |  | **Coordinates** | **Locus** | **Gene name or product** |
| **Ⅰ** | 460743-460744 (55bp) | MRA_0391 upstream | putative conserved secreted protein |  | 459381-459435 | Rv0383c upstream | POSSIBLE CONSERVED SECRETED PROTEIN |
|  | 3427350-3427351 (14bp) | MRA_3085 upstream | putative glutaredoxin NrdH |  | 3415181-3415194 | Rv3053c upstream | PROBABLE GLUTAREDOXIN ELECTRON  TRANSPORT COMPONENT OF NRDEF NRDH |
|  | 3591419-3591420 (1bp) | MRA_3241 upstream | lipase LipV |  | 3580637 | Rv3203 upstream | POSSIBLE LIPASE LIPV |
|  | 3871150-3871151 (1bp) | MRA_3484 upstream | 50S ribosomal protein L13 | yes | 3862473 | Rv3443c upstream | 50S ribosomal protein L13 |
|  | 3569552-3569553 (15bp) | MRA_3225 | hypothetical protein |  | 3558755-3558769 |  |  |
| **Ⅱ** | 3563382-3563383 (1358bp) |  |  | yes | 3551227-3552584 | Rv3184 | PROBABLE TRANSPOSASE |
|  |  |  |  |  |  | Rv3185 | PROBABLE TRANSPOSASE |
|  | 4061598-4061599 (106bp) | MRA_3649  upstream | cell division protein FtsH |  | 4052934-4053039 | Rv3610c  upstream | MEMBRANE-BOUND PROTEASE FTSH |
|  | 4061608-4061609 (82bp) |  |  |  | 4053050-4053131 |  |  |
|  | 4061612-4061613 (30bp) |  |  |  | 4053136-4053165 |  |  |
| **Ⅲ** | 338045-338046 (1bp) | MRA_0288 | PE-PGRS family protein |  | 336684 | Rv0279c | PE-PGRS FAMILY PROTEIN |
|  | 3957248-3957249 (240bp) | MRA_3553 | PE-PGRS family protein |  | 3947749-3947988 | Rv3514 | PE-PGRS FAMILY PROTEIN |
|  | 3958198-3958199 (603bp) |  |  |  | 3948930-3949532 |  |  |
|  | 2535631-2535632 (1bp) | MRA_2271 | putative flavoprotein | yes | 2525727 | Rv2250A | POSSIBLE FLAVOPROTEIN |
|  |  |  |  |  |  | Rv2251 | POSSIBLE FLAVOPROTEIN |
|  | 4103448-4103449 (1bp) | MRA_3690 | hypothetical protein | yes | 4095002 | Rv3655c | hypothetical protein Rv3655c |
|  | 4409106-4409107 (1bp) | MRA_3950 | RNA polymerase sigma-70 factor | yes | 4400661 | Rv3911 | RNA polymerase sigma-70 factor |
|  | 3741252-3741253 (1728bp) | MRA_3384 | PPE family protein |  | 3730412-3732139 | Rv3343c | PPE FAMILY PROTEIN |
|  | 3812095-3812096 (447bp) | MRA_3428 | PE-PGRS family protein |  | 3802973-3803419 | Rv3388 | PE-PGRS FAMILY PROTEIN |
|  | 733673-733674 (5bp) | MRA_0646 | hypothetical protein |  | 732364-732368 | Rv0635 | hypothetical protein Rv0635 |
|  | 1341581-1341582 (1bp) | MRA_1205A | PPE family protein |  | 1340271 | Rv1196 | PPE FAMILY PROTEIN |
|  | 2191301-2191302 (33bp) | MRA_1940 | hypothetical protein |  | 2181370-2181402 | Rv1929c | hypothetical protein Rv1929c |
|  | 4046228-4046229 (1bp) | MRA_3635 | PE-PGRS family protein |  | 4037563 | Rv3595c | PE-PGRS FAMILY PROTEIN |

Note: Five deletions occur in the intergenic regions of H37Rv (Part I of Table S2). Three deletions in Rv3611 render the disruption of this gene in H37Ra, and a 1358 bp deletion caused the disappearance of one IS6110 element at 3563 kb in H37Ra (Part II of Table S2). The remaining twelve deletions (Part III of Table S2) affect 11 H37Ra CDSs, most of which encode PE/PPE/PGRS family proteins.
