# Supplementary material for: Genetic Basis of Virulence Attenuation Revealed by Comparative Genomic Analysis of Mycobacterium tuberculosis Strain H37Ra versus H37Rv
Source: PLoS One. 2008 Jun 11;3(6):e2375. doi: 10.1371/journal.pone.0002375 (PMC2440308; doi:10.1371/journal.pone.0002375)
Supplement: Table S3 — (0.60 MB DOC) [file pone.0002375.s004.doc]

**Table S3. SNVs detected in H37Ra compared to H37Rv.**

| **SNVs in H37Ra** | | | **Nucleotide substitution** | **Amino acids change** | **SNVs same  as in**  **CDC1551** | **SNVs in H37Rv** | | |
| --- | --- | --- | --- | --- | --- | --- | --- | --- |
| **Site** | **Locus** | **Gene name or product** | **H37Ra←H37Rv** | **H37Ra←H37Rv** |  | **Site** | **Locus** | **Gene name or product** |
| 16143 | MRA_0014 | putative conserved membrane protein | c←t | Arg←Cys | yes | 14785 | Rv0012 | PROBABLE CONSERVED MEMBRANE PROTEIN |
| 42031 | MRA_0040 | putative conserved integral membrane protein | t←c | Asp←Gly |  | 40673 | Rv0037c | PROBABLE CONSERVED INTEGRAL MEMBRANE PROTEIN |
| 43775 | MRA_0042up | putative conserved transmembrane protein | c←t |  |  | 42417 | Rv0039cup | POSSIBLE CONSERVED TRANSMEMBRANE PROTEIN |
| 56911 | MRA_0053 | bifunctional penicillin-binding protein 1A/1B PonA1 | t←c | Ser←Pro | yes | 55553 | Rv0050 | PROBABLE BIFUNCTIONAL ENICILLIN- BINDING PROTEIN 1A/1B PONA1 |
| 71347 | MRA_0066 | hypothetical protein | a←g | Asp←Gly | yes | 69989 | Rv0064 | hypothetical protein Rv0064 |
| 91502 | MRA_0086 | putative oxidoreductase | g←a | Arg←Gln | yes | 90144 | Rv0082 | PROBABLE OXIDOREDUCTASE |
| 92429 | MRA_0087 | putative NADH-ubiquinone oxidoreductase | c←t | Ile←Ile | yes | 91071 | Rv0083 | PROBABLE OXIDOREDUCTASE |
| 111636 | MRA_0105 | putative peptide synthetase | a←g | *←Trp |  | 110278 | Rv0101 | PROBABLE PEPTIDE SYNTHETASE NRP |
| 117358 | MRA_0106 | putative peptide synthetase | g←t | Val←Val | yes | 116000 | Rv0101 | PROBABLE PEPTIDE SYNTHETASE NRP |
| 133776 | MRA_0115 | PE-PGRS family protein | g←c | Gly←Arg | yes | 132417 | Rv0109 | PE-PGRS FAMILY PROTEIN |
| 151991 | MRA_0131 | PE-PGRS family protein | t←c | Val←Ala |  | 150632 | Rv0124 | PE-PGRS FAMILY PROTEIN |
| 222233 | MRA_0197 | dihydroxy-acid dehydratase | c←a | Gly←Val |  | 220873 | Rv0189c | dihydroxy-acid dehydratase |
| 222319 | MRA_0197 | dihydroxy-acid dehydratase | c←a | Gly←Gly |  | 220959 | Rv0189c | dihydroxy-acid dehydratase |
| 235837 | MRA_0205 | putative oxidoreductase | g←t | *←Tyr | yes | 234477 | Rv0197 | POSSIBLE OXIDOREDUCTASE |
| 243661 | MRA_0212 | putative conserved transmembrane protein | g←c | Leu←Val | yes | 242299 | Rv0204c | PROBABLE CONSERVED TRANSMEMBRANE PROTEIN |
| 335254 | MRA_0287 | PE-PGRS family protein | c←g | Gly←Arg | yes | 333892 | Rv0278c | PE-PGRS FAMILY PROTEIN |
| 338043 | MRA_0288 | PE-PGRS family protein | c←g | Gly←Pro | yes | 336681 | Rv0279c | PE-PGRS FAMILY PROTEIN |
| 338044 | MRA_0288 | PE-PGRS family protein | c←g | Gly←Pro | yes | 336682 | Rv0279c | PE-PGRS FAMILY PROTEIN |
| 338050 | MRA_0288 | PE-PGRS family protein | c←g | Val←Ser | yes | 336689 | Rv0279c | PE-PGRS FAMILY PROTEIN |
| 338051 | MRA_0288 | PE-PGRS family protein | a←c | Gly←Ser |  | 336690 | Rv0279c | PE-PGRS FAMILY PROTEIN |
| 338052 | MRA_0288 | PE-PGRS family protein | c←t | Gly←Ser | yes | 336691 | Rv0279c | PE-PGRS FAMILY PROTEIN |
| 338055 | MRA_0288 | PE-PGRS family protein | c←a | Gly←Ser | yes | 336694 | Rv0279c | PE-PGRS FAMILY PROTEIN |
| 338059 | MRA_0288 | PE-PGRS family protein | a←t | Tyr←Asn |  | 336697 | Rv0279c | PE-PGRS FAMILY PROTEIN |
| 338060 | MRA_0288 | PE-PGRS family protein | a←c | Gly←Gly |  | 336698 | Rv0279c | PE-PGRS FAMILY PROTEIN |
| 338063 | MRA_0288 | PE-PGRS family protein | t←a | Gly←Gly |  | 336701 | Rv0279c | PE-PGRS FAMILY PROTEIN |
| 338069 | MRA_0288 | PE-PGRS family protein | a←g | Gly←Asp | yes | 336707 | Rv0279c | PE-PGRS FAMILY PROTEIN |
| 338070 | MRA_0288 | PE-PGRS family protein | c←t | Gly←Asp | yes | 336708 | Rv0279c | PE-PGRS FAMILY PROTEIN |
| 338072 | MRA_0288 | PE-PGRS family protein | g←a | Ala←Ala | yes | 336710 | Rv0279c | PE-PGRS FAMILY PROTEIN |
| 338102 | MRA_0288 | PE-PGRS family protein | t←g | Lys←Gly |  | 336740 | Rv0279c | PE-PGRS FAMILY PROTEIN |
| 338103 | MRA_0288 | PE-PGRS family protein | t←c | Lys←Gly |  | 336741 | Rv0279c | PE-PGRS FAMILY PROTEIN |
| 338104 | MRA_0288 | PE-PGRS family protein | t←c | Lys←Gly |  | 336742 | Rv0279c | PE-PGRS FAMILY PROTEIN |
| 338105 | MRA_0288 | PE-PGRS family protein | t←g | Lys←Asn |  | 336743 | Rv0279c | PE-PGRS FAMILY PROTEIN |
| 339321 | MRA_0288 | PE-PGRS family protein | c←a | Ser←Ile | yes | 337959 | Rv0279c | PE-PGRS FAMILY PROTEIN |
| 339382 | MRA_0288 | PE-PGRS family protein | c←a | Gly←Cys | yes | 338020 | Rv0279c | PE-PGRS FAMILY PROTEIN |
| 339462 | MRA_0288 | PE-PGRS family protein | c←t | Ser←Asn | yes | 338100 | Rv0279c | PE-PGRS FAMILY PROTEIN |
| 339815 | MRA_0288 | PE-PGRS family protein | g←a | Ala←Ala | yes | 338453 | Rv0279c | PE-PGRS FAMILY PROTEIN |
| 392190 | MRA_0332 | hypothetical protein | c←t | Gly←Ser |  | 390828 | Rv0323c | hypothetical protein Rv0323c |
| 428272 | MRA_0364 | PPE family protein | c←a | Gly←Trp | yes | 426909 | Rv0355c | PPE FAMILY PROTEIN |
| 459645 | MRA_0390 | orotate phosphoribosyltransferase | g←a | Tyr←Tyr | yes | 458282 | Rv0382c | orotate phosphoribosyltransferase |
| 468826 | MRA_0395 | PPE family protein | c←g | Ser←Ser | yes | 467516 | Rv0388c | PPE FAMILY PROTEIN |
| 468836 | MRA_0395 | PPE family protein | g←c | Ala←Gly | yes | 467526 | Rv0388c | PPE FAMILY PROTEIN |
| 468856 | MRA_0395 | PPE family protein | c←g | Glu←Asp | yes | 467546 | Rv0388c | PPE FAMILY PROTEIN |
| 468867 | MRA_0395 | PPE family protein | c←a | Val←Leu | yes | 467557 | Rv0388c | PPE FAMILY PROTEIN |
| 468874 | MRA_0395 | PPE family protein | c←a | Gln←His | yes | 467564 | Rv0388c | PPE FAMILY PROTEIN |
| 468895 | MRA_0395 | PPE family protein | c←g | Gln←His | yes | 467585 | Rv0388c | PPE FAMILY PROTEIN |
| 468900 | MRA_0395 | PPE family protein | c←t | Ala←Thr | yes | 467590 | Rv0388c | PPE FAMILY PROTEIN |
| 468931 | MRA_0395 | PPE family protein | g←t | Gly←Gly | yes | 467621 | Rv0388c | PPE FAMILY PROTEIN |
| 468948 | MRA_0395 | PPE family protein | t←g | Lys←Gln | yes | 467638 | Rv0388c | PPE FAMILY PROTEIN |
| 512828 | MRA_0430 | metal cation transporting P-type ATPase CtpH | g←t | Ile←Ile | yes | 511518 | Rv0425c | POSSIBLE METAL CATION RANSPORTING  P-TYPE ATPASE CTPH |
| 533407 | MRA_0447 | PPE family protein | c←t | Glu←Lys | yes | 532097 | Rv0442c | PPE FAMILY PROTEIN |
| 553395 | MRA_0467 | hypothetical protein | g←a | Gln←Gln | yes | 552085 | Rv0461 | PROBABLE TRANSMEMBRANE PROTEIN |
| 564887 | MRA_0480 | hypothetical protein | g←a | Arg←Lys | yes | 563577 | Rv0473 | POSSIBLE CONSERVED TRANSMEMBRANE PROTEIN |
| 624782 | MRA_0539 | PE-PGRS family protein | g←a | Gly←Asp | yes | 623472 | Rv0532 | PE-PGRS FAMILY PROTEIN |
| 624818 | MRA_0539 | PE-PGRS family protein | g←c | Gly←Ala | yes | 623508 | Rv0532 | PE-PGRS FAMILY PROTEIN |
| 673801 | MRA_0585 | PE-PGRS family protein | g←c | Gly←Gly | yes | 672491 | Rv0578c | PE-PGRS FAMILY PROTEIN |
| 676400 | MRA_0585 | PE-PGRS family protein | c←t | Arg←Glu |  | 675090 | Rv0578c | PE-PGRS FAMILY PROTEIN |
| 676401 | MRA_0585 | PE-PGRS family protein | t←c | Arg←Glu |  | 675091 | Rv0578c | PE-PGRS FAMILY PROTEIN |
| 755492 | MRA_0668 | putative conserved integral membrane protein | g←a | Pro←Leu | yes | 754186 | Rv0658c | PROBABLE CONSERVED INTEGRAL MEMBRANE PROTEIN |
| 783228 | MRA_0691 | 30S ribosomal protein S12 | g←a | Lys←Lys | yes | 781922 | Rv0682 | 30S ribosomal protein S12 |
| 837578 | MRA_0754 | PE-PGRS family protein | g←a | Gly←Glu | yes | 836272 | Rv0746 | PE-PGRS FAMILY PROTEIN |
| 837597 | MRA_0754 | PE-PGRS family protein | g←a | Gly←Gly | yes | 836291 | Rv0746 | PE-PGRS FAMILY PROTEIN |
| 837732 | MRA_0754 | PE-PGRS family protein | c←a | Leu←Leu | yes | 836426 | Rv0746 | PE-PGRS FAMILY PROTEIN |
| 837760 | MRA_0754 | PE-PGRS family protein | g←a | Ala←Thr | yes | 836454 | Rv0746 | PE-PGRS FAMILY PROTEIN |
| 837844 | MRA_0754 | PE-PGRS family protein | g←a | Asp←Asn | yes | 836538 | Rv0746 | PE-PGRS FAMILY PROTEIN |
| 837964 | MRA_0754 | PE-PGRS family protein | g←a | Ala←Thr | yes | 836658 | Rv0746 | PE-PGRS FAMILY PROTEIN |
| 838339 | MRA_0754 | PE-PGRS family protein | g←a | Ala←Thr | yes | 837033 | Rv0746 | PE-PGRS FAMILY PROTEIN |
| 840296 | MRA_0755 | PE-PGRS family protein | g←c | Ala←Ala | yes | 838990 | Rv0747 | PE-PGRS FAMILY PROTEIN |
| 840429 | MRA_0755 | PE-PGRS family protein | g←a | Gly←Arg | yes | 839123 | Rv0747 | PE-PGRS FAMILY PROTEIN |
| 840435 | MRA_0755 | PE-PGRS family protein | g←c | Gly←Arg | yes | 839129 | Rv0747 | PE-PGRS FAMILY PROTEIN |
| 840500 | MRA_0755 | PE-PGRS family protein | g←a | Thr←Thr | yes | 839194 | Rv0747 | PE-PGRS FAMILY PROTEIN |
| 840640 | MRA_0755 | PE-PGRS family protein | g←a | Arg←Lys | yes | 839334 | Rv0747 | PE-PGRS FAMILY PROTEIN |
| 840654 | MRA_0755 | PE-PGRS family protein | g←a | Gly←Ser | yes | 839348 | Rv0747 | PE-PGRS FAMILY PROTEIN |
| 841802 | MRA_0755 | PE-PGRS family protein | g←c | Gly←Gly | yes | 840496 | Rv0747 | PE-PGRS FAMILY PROTEIN |
| 853569 | MRA_0767 | putative two component system response ranscriptional positive regulator PhoP | t←c | Leu←Ser |  | 852263 | Rv0757 | POSSIBLE TWO COMPONENT SYSTEM RESPONSE TRANSCRIPTIONAL POSITIVE REGULATOR PHOP |
| 928416 | MRA_0841a | PE-PGRS family protein | g←a | Gly←Ser | yes | 927110 | Rv0833 | PE-PGRS FAMILY PROTEIN |
| 980440 | MRA_0887 | putative marr-family transcriptional  regulatory protein | a←g | Lys←Arg |  | 979133 | Rv0880 | POSSIBLE TRANSCRIPTIONAL REGULATORY PROTEIN (POSSIBLY MARR-FAMILY) |
| 980441 | MRA_0887 | putative marr-family transcriptional  regulatory protein | a←g | Lys←Arg |  | 979134 | Rv0880 | POSSIBLE TRANSCRIPTIONAL REGULATORY PROTEIN (POSSIBLY MARR-FAMILY) |
| 991308 | MRA_0898 | LuxR family transcriptional regulator | c←g | Ala←Pro | yes | 990001 | Rv0890c | PROBABLE TRANSCRIPTIONAL REGULATORY PROTEIN (PROBABLY LUXR-FAMILY) |
| 1026414 | MRA_0927 | hypothetical protein | c←t | Phe←Phe | yes | 1025106 | Rv0919 | hypothetical protein Rv0919 |
| 1039219 | MRA_0938 | phosphate ABC transporter permease  protein PstA1 | t←c | *←Arg | yes | 1037911 | Rv0930 | PROBABLE PHOSPHATE-TRANSPORT INTEGRAL MEMBRANE ABC TRANSPORTER PSTA1 |
| 1078620 | MRA_0973 | hypothetical protein | g←a | Ala←Val | yes | 1077312 | Rv0966c | hypothetical protein Rv0966c |
| 1092860 | MRA_0984 | PE-PGRS family protein | t←g | Ser←Ala |  | 1091552 | Rv0977 | PE-PGRS FAMILY PROTEIN |
| 1092891 | MRA_0984 | PE-PGRS family protein | t←g | Val←Gly |  | 1091583 | Rv0977 | PE-PGRS FAMILY PROTEIN |
| 1094714 | MRA_0985 | PE-PGRS family protein | g←a | Val←Val | yes | 1093406 | Rv0978c | PE-PGRS FAMILY PROTEIN |
| 1124972 | MRA_1014up | para-aminobenzoate synthase component I | a←t |  |  | 1123664 | Rv1005cup | para-aminobenzoate synthase component I |
|  | MRA1015up | hypothetical protein |  |  |  |  | Rv1006up | hypothetical protein Rv1006 |
| 1144634 | MRA_1029 | conserved hypothetical protein | a←c | Glu←Ala |  | 1143326 | Rv1021 | hypothetical protein Rv1021 |
| 1192696 | MRA_1078 | PE-PGRS family protein | t←g | Gly←Gly |  | 1191387 | Rv1068c | PE-PGRS FAMILY PROTEIN |
| 1192748 | MRA_1078 | PE-PGRS family protein | a←c | Val←Gly |  | 1191439 | Rv1068c | PE-PGRS FAMILY PROTEIN |
| 1219188 | MRA_1102 | PE-PGRS family protein | a←g | Ser←Gly |  | 1217879 | Rv1091 | PE-PGRS FAMILY PROTEIN |
| 1224107 | MRA_1106up | putative PhoH-like protein PhoH2 | a←g |  |  | 1222797 | Rv1095up | PROBABLE PHOH-LIKE PROTEIN PHOH2 |
| 1246010 | MRA_1130 | putative glucose-6-phosphate 1-dehydrogenase Zwf1 | c←t | Leu←Leu | yes | 1244700 | Rv1121 | glucose-6-phosphate 1-dehydrogenase |
| 1314648 |  |  | g←a |  | yes | 1313338 |  |  |
| 1316502 | MRA_1191 | polyketide beta-ketoacyl synthase Pks3 | c←a | Ser←* | yes | 1315191 | Rv1180 | PROBABLE POLYKETIDE BETA-KETOACYL  SYNTHASE PKS3 |
| 1317195 | MRA_1191 | polyketide beta-ketoacyl synthase Pks3 | a←g | Ala←Ala | yes | 1315884 | Rv1181 | PROBABLE POLYKETIDE BETA-KETOACYL  SYNTHASE PKS4 |
| 1328713 | MRA_1195 | fatty-acid-CoA ligase FadD21 | c←t | Glu←Glu | yes | 1327402 | Rv1185c | acyl-CoA synthase |
| 1333007 | MRA_1198 | proline dehydrogenase family protein | c←a | Arg←Arg | yes | 1331696 | Rv1188 | PROBABLE PROLINE DEHYDROGENASE |
| 1415331 | MRA_1274 | serine/threonine protein kinase | t←c | Gln←Arg | yes | 1414021 | Rv1266c | PROBABLE TRANSMEMBRANE SERINE/THREONINE-PROTEIN KINASE H PKNH |
| 1454918 | MRA_1305 | transcription termination factor Rho | c←t | Gly←Gly | yes | 1453608 | Rv1297 | transcription termination factor Rho |
| 1472969 |  |  | t←c |  | yes | 1471659 |  |  |
| 1562654 | MRA_1395up | PE family protein | g←a |  |  | 1561344 | Rv1386up | PE FAMILY PROTEIN |
| 1618030 |  |  | c←g |  |  | 1616720 |  |  |
| 1619492 |  |  | t←g |  |  | 1618182 |  |  |
| 1634199 | MRA_1459 | PE-PGRS family protein | a←c | Ile←Ser |  | 1632889 | Rv1450c | PE-PGRS FAMILY PROTEIN |
| 1903334 | MRA_1688 | putative lipoprotein DsbF | g←a | Gln←Gln | yes | 1901816 | Rv1677 | PROBABLE CONSERVED LIPOPROTEIN DSBF |
| 2030495 | MRA_1798 | FtsK/SpoIIIE family protein | t←a | Leu←* | yes | 2020563 | Rv1783 | PROBABLE CONSERVED MEMBRANE PROTEIN |
| 2053216 | MRA_1815up | PPE family protein | t←g |  |  | 2043284 | Rv1802up | PPE FAMILY PROTEIN |
| 2060845 | MRA_1820 | PPE family protein | g←a | Glu←Glu | yes | 2050913 | Rv1808 | PPE FAMILY PROTEIN |
| 2061678 | MRA_1821 | PPE family protein | c←t | Ala←Ala |  | 2051746 | Rv1809 | PPE FAMILY PROTEIN |
| 2067706 | MRA_1827 | hypothetical protein | t←a | Phe←Ile | yes | 2057774 | Rv1815 | hypothetical protein Rv1815 |
| 2177421 |  |  | c←t |  | yes | 2167489 |  |  |
| 2187586 | MRA_1936 | fatty-acid-CoA ligase FadD31 | c←a | Leu←Met | yes | 2177654 | Rv1925 | acyl-CoA synthase |
| 2231696 | MRA_1990 | amino acid permease | t←c | Ile←Val | yes | 2221796 | Rv1979c | POSSIBLE CONSERVED PERMEASE |
| 2261899 |  |  | g←a |  | yes | 2251999 |  |  |
| 2292687 | MRA_2052 | hypothetical protein | t←c | Tyr←Cys | yes | 2282787 | Rv2037c | POSSIBLE CONSERVED TRANSMEMBRANE  PROTEIN |
| 2307876 | MRA_2063 | polyketide synthase Pks12 | a←g | Leu←Ser | yes | 2297976 | Rv2048c | Probable polyketide synthase pks12 |
| 2313721 | MRA_2063 | polyketide synthase Pks12 | c←g | Ala←Pro |  | 2303821 | Rv2048c | Probable polyketide synthase pks12 |
| 2336793 | MRA_2082up | beta-lactamase | t←a |  |  | 2326893 | Rv2068cup | CLASS A BETA-LACTAMASE BLAC |
|  | MRA_2083up | RNA polymerase sigma-70 factor |  |  |  |  | Rv2069up | RNA polymerase sigma-70 factor |
| 2371525 | MRA_2115 | putative helicase HelZ | c←a | Leu←Met | yes | 2361623 | Rv2101 | PROBABLE HELICASE HELZ |
| 2397635 | MRA_2140a | PE-PGRS family protein | c←t | Glu←Glu | yes | 2387733 | Rv2126c | PE-PGRS FAMILY PROTEIN |
| 2477033 | MRA_2218 | carbohydrate kinase CbhK | a←g | Asp←Asp |  | 2467131 | Rv2202c | Probable carbohydrate kinase CbhK |
| 2480051 | MRA_2221 | hypothetical protein | c←t | Glu←Glu | yes | 2470149 | Rv2205c | hypothetical protein Rv2205c |
| 2515821 | MRA_2251 | hypothetical protein | g←a |  | yes | 2505919 |  |  |
| 2705335 | MRA_2420 | PE-PGRS family protein | g←a | Gly←Asp |  | 2693367 | Rv2396 | PE-PGRS FAMILY PROTEIN |
| 2730820 | MRA_2447up | nicotinic acid mononucleotide adenyltransferase | g←t |  |  | 2718852 | Rv2421cup | nicotinic acid mononucleotide adenyltransferase |
| 2763772 | MRA_2476 | putative resuscitation-promoting factor RpfE | t←c | Gln←Arg | yes | 2751804 | Rv2450c | PROBABLE RESUSCITATION-PROMOTING  FACTOR RPFE |
| 2821589 | MRA_2521 | dihydrolipoamide S-acetyltransferase E2 component PdhC | c←t | Ala←Thr | yes | 2809621 | Rv2495c | dihydrolipoamide acetyltransferase |
| 2955379 |  |  | c←t |  | yes | 2943411 | Rv2614A | hypothetical protein Rv2614A |
| 2966407 | MRA_2655 | hypothetical protein | c←t | Gly←Arg | yes | 2954439 | Rv2627c | hypothetical protein Rv2627c |
| 2984929 | MRA_2678 | IS6110 transposase | g←c | Gly←Ala |  | 2972961 | Rv2649 | PROBABLE TRANSPOSASE FOR INSERTION  SEQUENCE ELEMENT IS6110 |
| 3008162 | MRA_2708 | hypothetical protein | a←t | Val←Val | yes | 2996194 | Rv2680 | hypothetical protein Rv2680 |
| 3024261 | MRA_2723 | conserved hypothetical alanine rich protein | g←a | Thr←Thr | yes | 3012293 | Rv2695 | CONSERVED HYPOTHETICAL ALANINE RICH  PROTEIN |
| 3058627 | MRA_2759up | conserved hypothetical alanine and arginine rich protein | g←a |  |  | 3046659 | Rv2733cup | CONSERVED HYPOTHETICAL ALANINE, ARGININE-RICH PROTEIN |
|  | MRA_2760up | hypothetical protein |  |  |  |  | Rv2734up | hypothetical protein Rv2734 |
| 3066692 | MRA_2767 | PE-PGRS family protein | g←a | Gly←Ser | yes | 3054724 | Rv2741 | PE-PGRS FAMILY PROTEIN |
| 3135120 |  |  | c←g |  |  | 3123146 |  |  |
| 3135127 |  |  | c←t |  |  | 3123151 |  |  |
| 3135170 |  |  | c←t |  |  | 3123185 |  |  |
| 3217970 | MRA_2921 | conserved hypothetical protein | c←a | Ala←Ser | yes | 3205978 | Rv2896c | hypothetical protein Rv2896c |
| 3266357 | MRA_2958 | phenolpthiocerol synthesis type-I polyketide synthase PpsB | c←t | Leu←Leu | yes | 3254365 | Rv2932 | PHENOLPTHIOCEROL SYNTHESIS TYPE-I POLYKETIDE SYNTHASE PPSB |
| 3378001 |  |  | a←c |  |  | 3365832 |  |  |
| 3382346 | MRA_3041 | glutamyl-tRNA amidotransferase subunit A | g←t | Leu←Met | yes | 3370177 | Rv3011c | glutamyl-tRNA amidotransferase subunit A |
| 3391877 | MRA_3052 | PPE family protein | c←g | Val←Leu | yes | 3379708 | Rv3021c | PPE FAMILY PROTEIN |
| 3391881 | MRA_3052 | PPE family protein | c←g | Ala←Ala | yes | 3379712 | Rv3021c | PPE FAMILY PROTEIN |
| 3391887 | MRA_3052 | PPE family protein | c←t | Gly←Gly | yes | 3379718 | Rv3021c | PPE FAMILY PROTEIN |
| 3391895 | MRA_3052 | PPE family protein | a←c | Leu←Val | yes | 3379726 | Rv3021c | PPE FAMILY PROTEIN |
| 3391899 | MRA_3052 | PPE family protein | c←g | Thr←Ala | yes | 3379730 | Rv3021c | PPE FAMILY PROTEIN |
| 3391901 | MRA_3052 | PPE family protein | t←c | Thr←Ala | yes | 3379732 | Rv3021c | PPE FAMILY PROTEIN |
| 3391904 | MRA_3052 | PPE family protein | c←a | Val←Leu | yes | 3379735 | Rv3021c | PPE FAMILY PROTEIN |
| 3391905 | MRA_3052 | PPE family protein | a←c | Gly←Gly | yes | 3379736 | Rv3021c | PPE FAMILY PROTEIN |
| 3391911 | MRA_3052 | PPE family protein | c←t | Val←Val | yes | 3379742 | Rv3021c | PPE FAMILY PROTEIN |
| 3391920 | MRA_3052 | PPE family protein | c←a | Val←Val | yes | 3379751 | Rv3021c | PPE FAMILY PROTEIN |
| 3391926 | MRA_3052 | PPE family protein | c←a | Ala←Ala | yes | 3379757 | Rv3021c | PPE FAMILY PROTEIN |
| 3391932 | MRA_3052 | PPE family protein | a←g | Ala←Ala | yes | 3379763 | Rv3021c | PPE FAMILY PROTEIN |
| 3391953 | MRA_3052 | PPE family protein | a←c | Gly←Gly | yes | 3379784 | Rv3021c | PPE FAMILY PROTEIN |
| 3391957 | MRA_3052 | PPE family protein | g←c | Ala←Gly | yes | 3379788 | Rv3021c | PPE FAMILY PROTEIN |
| 3402622 | MRA_3062 | hypothetical protein | a←g | Arg←Arg |  | 3390452 | Rv3031 | hypothetical protein Rv3031 |
| 3522798 | MRA_3177 | PPE family protein | c←t | Gly←Ser | yes | 3510642 | Rv3144c | PPE-FAMILY PROTEIN |
| 3729198 | MRA_3373 | sugar-transport integral membrane protein SugI | t←c | Leu←Pro | yes | 3718357 | Rv3331 | PROBABLE SUGAR-TRANSPORT INTEGRAL  MEMBRANE PROTEIN SUGI |
| 3812623 | MRA_3428A | hypothetical protein | a←t | Ser←Asp |  | 3803947 | Rv3389c | POSSIBLE DEHYDROGENASE |
| 3812626 | MRA_3428A | hypothetical protein | c←t | Ala←Asp |  | 3803950 | Rv3389c | POSSIBLE DEHYDROGENASE |
| 3812638 | MRA_3428A | hypothetical protein | a←c | Cys←Val |  | 3803960 | Rv3389c | POSSIBLE DEHYDROGENASE |
| 3905017 | MRA_3519 | hypothetical protein | g←t | Arg←Leu | yes | 3896340 | Rv3479 | POSSIBLE TRANSMEMBRANE PROTEIN |
| 3936806 | MRA_3547 | PE-PGRS family protein | c←a | His←Asn |  | 3928129 | Rv3507 | PE-PGRS FAMILY PROTEIN |
| 3944012 | MRA_3548 | PE-PGRS family protein | c←t | Ala←Val | yes | 3935335 | Rv3508 | PE-PGRS FAMILY PROTEIN |
| 3944668 |  |  | c←a |  |  | 3935426 | Rv3508 | PE-PGRS FAMILY PROTEIN |
| 3944680 |  |  | c←t |  |  | 3935431 | Rv3508 | PE-PGRS FAMILY PROTEIN |
| 3944767 |  |  | c←g |  | yes | 3935441 | Rv3508 | PE-PGRS FAMILY PROTEIN |
| 3944985 |  |  | a←c |  | yes | 3935494 | Rv3508 | PE-PGRS FAMILY PROTEIN |
| 3950293 | MRA_3551 | PE-PGRS family protein | g←a | Asp←Asn | yes | 3940802 | Rv3511 | PE-PGRS FAMILY PROTEIN |
| 3955465 | MRA_3553 | PE-PGRS family protein | c←t | Leu←Phe |  | 3945965 | Rv3514 | PE-PGRS FAMILY PROTEIN |
| 3956619 | MRA_3553 | PE-PGRS family protein | g←a | Gln←Gln |  | 3947119 | Rv3514 | PE-PGRS FAMILY PROTEIN |
| 3957463 | MRA_3553 | PE-PGRS family protein | g←a | Val←Ile |  | 3948203 | Rv3514 | PE-PGRS FAMILY PROTEIN |
| 3957616 | MRA_3553 | PE-PGRS family protein | g←t | Ala←Ser |  | 3948347 | Rv3514 | PE-PGRS FAMILY PROTEIN |
| 3957631 | MRA_3553 | PE-PGRS family protein | g←t | Gly←Cys |  | 3948362 | Rv3514 | PE-PGRS FAMILY PROTEIN |
| 3957640 | MRA_3553 | PE-PGRS family protein | a←g | Ser←Gly |  | 3948371 | Rv3514 | PE-PGRS FAMILY PROTEIN |
| 3957673 | MRA_3553 | PE-PGRS family protein | g←t | Gly←Cys |  | 3948404 | Rv3514 | PE-PGRS FAMILY PROTEIN |
| 3957683 | MRA_3553 | PE-PGRS family protein | g←t | Gly←Val |  | 3948414 | Rv3514 | PE-PGRS FAMILY PROTEIN |
| 3957686 | MRA_3553 | PE-PGRS family protein | g←t | Gly←Val |  | 3948417 | Rv3514 | PE-PGRS FAMILY PROTEIN |
| 3958243 | MRA_3553 | PE-PGRS family protein | g←a | Ala←Thr |  | 3949577 | Rv3514 | PE-PGRS FAMILY PROTEIN |
| 3958247 | MRA_3553 | PE-PGRS family protein | t←a | Ile←Asn |  | 3949581 | Rv3514 | PE-PGRS FAMILY PROTEIN |
| 3958274 | MRA_3553 | PE-PGRS family protein | t←g | Val←Gly |  | 3949608 | Rv3514 | PE-PGRS FAMILY PROTEIN |
| 3958275 | MRA_3553 | PE-PGRS family protein | c←a | Val←Gly |  | 3949609 | Rv3514 | PE-PGRS FAMILY PROTEIN |
| 3958279 | MRA_3553 | PE-PGRS family protein | t←g | Cys←Gly |  | 3949613 | Rv3514 | PE-PGRS FAMILY PROTEIN |
| 3958316 | MRA_3553 | PE-PGRS family protein | t←g | Val←Gly |  | 3949650 | Rv3514 | PE-PGRS FAMILY PROTEIN |
| 3958365 | MRA_3553 | PE-PGRS family protein | g←c | Gly←Gly |  | 3949699 | Rv3514 | PE-PGRS FAMILY PROTEIN |
| 3958367 | MRA_3553 | PE-PGRS family protein | g←a | Gly←Asp |  | 3949701 | Rv3514 | PE-PGRS FAMILY PROTEIN |
| 3958376 | MRA_3553 | PE-PGRS family protein | t←a | Ile←Asn |  | 3949710 | Rv3514 | PE-PGRS FAMILY PROTEIN |
| 3958394 | MRA_3553 | PE-PGRS family protein | c←g | Ala←Gly |  | 3949728 | Rv3514 | PE-PGRS FAMILY PROTEIN |
| 3958427 | MRA_3553 | PE-PGRS family protein | c←g | Ala←Gly |  | 3949761 | Rv3514 | PE-PGRS FAMILY PROTEIN |
| 3958450 | MRA_3553 | PE-PGRS family protein | t←g | Ser←Ala |  | 3949784 | Rv3514 | PE-PGRS FAMILY PROTEIN |
| 3958474 | MRA_3553 | PE-PGRS family protein | c←t | Leu←Phe |  | 3949808 | Rv3514 | PE-PGRS FAMILY PROTEIN |
| 3958477 | MRA_3553 | PE-PGRS family protein | t←a | Tyr←Asn |  | 3949811 | Rv3514 | PE-PGRS FAMILY PROTEIN |
| 3958515 | MRA_3553 | PE-PGRS family protein | a←c | Thr←Thr |  | 3949849 | Rv3514 | PE-PGRS FAMILY PROTEIN |
| 3958516 | MRA_3553 | PE-PGRS family protein | a←g | Ser←Gly |  | 3949850 | Rv3514 | PE-PGRS FAMILY PROTEIN |
| 4061613 | MRA_3649up | cell division protein FtsH | t←c |  |  | 4053166 | Rv3610cup | MEMBRANE-BOUND PROTEASE FTSH |
| 4109421 |  |  | c←t |  | yes | 4100975 |  |  |
| 4155516 | MRA_3740 | putative glutamate--cysteine ligase | g←a | Leu←Leu | yes | 4147070 | Rv3704c | GLUTAMATE--CYSTEINE LIGASE GSHA (GAMMA-GLUTAMYLCYSTEINE SYNTHETASE) |
| 4367545 | MRA_3918 | hypothetical alanine and proline rich protein | c←g | Met←Ile |  | 4359099 | Rv3879c | HYPOTHETICAL ALANINE AND PROLINE  RICH PROTEIN |
| 4416349 | MRA_3958 | glucose-inhibited division protein B | a←g | Phe←Ser | yes | 4407904 | Rv3919c | glucose-inhibited division protein B |
