# Supplementary material for: Genetic Basis of Virulence Attenuation Revealed by Comparative Genomic Analysis of Mycobacterium tuberculosis Strain H37Ra versus H37Rv
Source: PLoS One. 2008 Jun 11;3(6):e2375. doi: 10.1371/journal.pone.0002375 (PMC2440308; doi:10.1371/journal.pone.0002375)
Supplement: Table S4 — (0.34 MB DOC) [file pone.0002375.s005.doc]

**Table S4. Re-sequencing results for the H37Rv variation sites**

| Re-sequencing results of H37Rv-specific SNVs | | | | | | |
| --- | --- | --- | --- | --- | --- | --- |
| H37Ra position | H37Ra site | H37Rv position | H37Rv site^1^ | H37Rv re-sequencing^2^ | Locus_tag | Coding product |
| 16143 | c | 14785 | t | c | Rv0012 | membrane protein |
| 56911 | t | 55553 | c | t | Rv0050 | penicillin-binding protein 1A/1B PonA1 |
| 71347 | a | 69989 | g | a | Rv0064 | hypothetical protein |
| 91502 | g | 90144 | a | g | Rv0082 | oxidoreductase |
| 92429 | c | 91071 | t | c | Rv0083 | NADH-ubiquinone oxidoreductase |
| 117358 | g | 116000 | t | g | Rv0101 | peptide synthetase |
| 133776 | g | 132417 | c | g | Rv0109 | PE-PGRS family protein |
| 235837 | g | 234477 | t | g | Rv0197 | oxidoreductase |
| 243661 | g | 242299 | c | g | Rv0204c | transmembrane protein |
| 335254 | c | 333892 | g | ? | Rv0278c | PE-PGRS family protein |
| 338043 | c | 336681 | g | ? | Rv0279c | PE-PGRS family protein |
| 338044 | c | 336682 | g | ? | Rv0279c | PE-PGRS family protein |
| 338050 | c | 336689 | g | ? | Rv0279c | PE-PGRS family protein |
| 338052 | c | 336691 | t | ? | Rv0279c | PE-PGRS family protein |
| 338055 | c | 336694 | a | ? | Rv0279c | PE-PGRS family protein |
| 338069 | a | 336707 | g | ? | Rv0279c | PE-PGRS family protein |
| 338070 | c | 336708 | t | ? | Rv0279c | PE-PGRS family protein |
| 338072 | g | 336710 | a | ? | Rv0279c | PE-PGRS family protein |
| 339321 | c | 337959 | a | ? | Rv0279c | PE-PGRS family protein |
| 339382 | c | 338020 | a | ? | Rv0279c | PE-PGRS family protein |
| 339462 | c | 338100 | t | ? | Rv0279c | PE-PGRS family protein |
| 339815 | g | 338453 | a | ? | Rv0279c | PE-PGRS family protein |
| 428272 | c | 426909 | a | c | Rv0355c | PPE family protein |
| 459645 | g | 458282 | a | g | Rv0382c | orotate phosphoribosyltransferase |
| 468826 | c | 467516 | g | c | Rv0388c | PPE family protein |
| 468836 | g | 467526 | c | g | Rv0388c | PPE family protein |
| 468856 | c | 467546 | g | c | Rv0388c | PPE family protein |
| 468867 | c | 467557 | a | c | Rv0388c | PPE family protein |
| 468874 | c | 467564 | a | c | Rv0388c | PPE family protein |
| 468895 | c | 467585 | g | c | Rv0388c | PPE family protein |
| 468900 | c | 467590 | t | c | Rv0388c | PPE family protein |
| 468931 | g | 467621 | t | g | Rv0388c | PPE family protein |
| 468948 | t | 467638 | g | t | Rv0388c | PPE family protein |
| 512828 | g | 511518 | t | g | Rv0425c | metal cation transporting P-type ATPase CtpH |
| 533407 | c | 532097 | t | c | Rv0442c | PPE family protein |
| 553395 | g | 552085 | a | g | Rv0461 | hypothetical protein |
| 564887 | g | 563577 | a | g | Rv0473 | hypothetical protein |
| 624782 | g | 623472 | a | ? | Rv0532 | PE-PGRS family protein |
| 624818 | g | 623508 | c | ? | Rv0532 | PE-PGRS family protein |
| 673801 | g | 672491 | c | ? | Rv0578c | PE-PGRS family protein |
| 755492 | g | 754186 | a | a | Rv0658c | integral membrane protein |
| 783228 | g | 781922 | a | g | Rv0682 | 30S ribosomal protein S12 |
| 837578 | g | 836272 | a | ? | Rv0746 | PE-PGRS family protein |
| 837597 | g | 836291 | a | ? | Rv0746 | PE-PGRS family protein |
| 837732 | c | 836426 | a | ? | Rv0746 | PE-PGRS family protein |
| 837760 | g | 836454 | a | ? | Rv0746 | PE-PGRS family protein |
| 837844 | g | 836538 | a | ? | Rv0746 | PE-PGRS family protein |
| 837964 | g | 836658 | a | ? | Rv0746 | PE-PGRS family protein |
| 838339 | g | 837033 | a | ? | Rv0746 | PE-PGRS family protein |
| 840296 | g | 838990 | c | ? | Rv0747 | PE-PGRS family protein |
| 840429 | g | 839123 | a | ? | Rv0747 | PE-PGRS family protein |
| 840435 | g | 839129 | c | ? | Rv0747 | PE-PGRS family protein |
| 840500 | g | 839194 | a | ? | Rv0747 | PE-PGRS family protein |
| 840640 | g | 839334 | a | ? | Rv0747 | PE-PGRS family protein |
| 840654 | g | 839348 | a | ? | Rv0747 | PE-PGRS family protein |
| 841802 | g | 840496 | c | ? | Rv0747 | PE-PGRS family protein |
| 928416 | g | 927110 | a | ? | Rv0833 | PE-PGRS family protein |
| 991308 | c | 990001 | g | c | Rv0890c | LuxR family transcriptional regulator |
| 1026414 | c | 1025106 | t | c | Rv0919 | hypothetical protein |
| 1039219 | t | 1037911 | c | t | Rv0930 | phosphate ABC transporter permease protein PstA1 |
| 1078620 | g | 1077312 | a | a | Rv0966c | hypothetical protein |
| 1094714 | g | 1093406 | a | ? | Rv0978c | PE-PGRS family protein |
| 1246010 | c | 1244700 | t | c | Rv1121 | glucose-6-phosphate 1-dehydrogenase Zwf1 |
| 1314648 | g | 1313338 | a | ? |  |  |
| 1316502 | c | 1315191 | a | ? | Rv1180 | polyketide beta-ketoacyl synthase Pks3 |
| 1317195 | a | 1315884 | g | a | Rv1181 | polyketide beta-ketoacyl synthase Pks3 |
| 1328713 | c | 1327402 | t | c | Rv1185c | fatty-acid-CoA ligase FadD21 |
| 1333007 | c | 1331696 | a | c | Rv1188 | proline dehydrogenase family protein |
| 1415331 | t | 1414021 | c | t | Rv1266c | serine/threonine protein kinase |
| 1454918 | c | 1453608 | t | c | Rv1297 | transcription termination factor Rho |
| 1472969 | t | 1471659 | c | t |  |  |
| 1903334 | g | 1901816 | a | g | Rv1677 | lipoprotein DsbF |
| 2030495 | t | 2020563 | a | t | Rv1783 | FtsK/SpoIIIE family protein |
| 2060845 | g | 2050913 | a | g | Rv1808 | PPE family protein |
| 2067706 | t | 2057774 | a | t | Rv1815 | hypothetical protein |
| 2177421 | c | 2167489 | t | ? |  |  |
| 2187586 | c | 2177654 | a | c | Rv1925 | fatty-acid-CoA ligase FadD31 |
| 2231696 | t | 2221796 | c | t | Rv1979c | amino acid permease |
| 2261899 | g | 2251999 | a | g |  |  |
| 2292687 | t | 2282787 | c | t | Rv2037c | hypothetical protein |
| 2307876 | a | 2297976 | g | a | Rv2048c | polyketide synthase Pks12 |
| 2371525 | c | 2361623 | a | c | Rv2101 | helicase HelZ |
| 2397635 | c | 2387733 | t | ? | Rv2126c | PE-PGRS family protein |
| 2480051 | c | 2470149 | t | c | Rv2205c | hypothetical protein |
| 2515821 | g | 2505919 | a | g |  |  |
| 2763772 | t | 2751804 | c | t | Rv2450c | resuscitation-promoting factor RpfE |
| 2821589 | c | 2809621 | t | c | Rv2495c | dihydrolipoamide S-acetyltransferase E2 component PdhC |
| 2955379 | c | 2943411 | t | ？ | Rv2614A | hypothetical protein |
| 2966407 | c | 2954439 | t | ？ | Rv2627c | hypothetical protein |
| 3008162 | a | 2996194 | t | a | Rv2680 | hypothetical protein |
| 3024261 | g | 3012293 | a | ？ | Rv2695 | alanine rich protein |
| 3066692 | g | 3054724 | a | ？ | Rv2741 | PE-PGRS family protein |
| 3217970 | c | 3205978 | a | c | Rv2896c | hypothetical protein |
| 3266357 | c | 3254365 | t | c | Rv2932 | phenolpthiocerol synthesis type-I polyketide synthase PpsB |
| 3382346 | g | 3370177 | t |  | Rv3011c | glutamyl-tRNA amidotransferase subunit A |
| 3391877 | c | 3379708 | g | c | Rv3021c | PPE family protein |
| 3391881 | c | 3379712 | g | c | Rv3021c | PPE family protein |
| 3391887 | c | 3379718 | t | c | Rv3021c | PPE family protein |
| 3391895 | a | 3379726 | c | a | Rv3021c | PPE family protein |
| 3391899 | c | 3379730 | g | c | Rv3021c | PPE family protein |
| 3391901 | t | 3379732 | c | t | Rv3021c | PPE family protein |
| 3391904 | c | 3379735 | a | c | Rv3021c | PPE family protein |
| 3391905 | a | 3379736 | c | a | Rv3021c | PPE family protein |
| 3391911 | c | 3379742 | t | c | Rv3021c | PPE family protein |
| 3391920 | c | 3379751 | a | c | Rv3021c | PPE family protein |
| 3391926 | c | 3379757 | a | c | Rv3021c | PPE family protein |
| 3391932 | a | 3379763 | g | a | Rv3021c | PPE family protein |
| 3391953 | a | 3379784 | c | a | Rv3021c | PPE family protein |
| 3391957 | g | 3379788 | c | g | Rv3021c | PPE family protein |
| 3522798 | c | 3510642 | t | ？ | Rv3144c | PPE family protein |
| 3729198 | t | 3718357 | c | ？ | Rv3331 | sugar-transport integral membrane protein SugI |
| 3905017 | g | 3896340 | t | g | Rv3479 | hypothetical protein |
| 3944012 | c | 3935335 | t | ？ | Rv3508 | PE-PGRS family protein |
| 3944767 | c | 3935441 | g | ？ | Rv3508 | PE-PGRS family protein |
| 3944985 | a | 3935494 | c | ？ | Rv3508 | PE-PGRS family protein |
| 3950293 | g | 3940802 | a | ？ | Rv3511 | PE-PGRS family protein |
| 4109421 | c | 4100975 | t | t |  |  |
| 4155516 | g | 4147070 | a | g | Rv3704c | glutamate--cysteine ligase |
| 4416349 | a | 4407904 | g | a | Rv3919c | glucose-inhibited division protein B |
| Re-sequencing results of 3 H37Ra-specific SNVs | | | | | | |
| H37Ra position | H37Ra site | H37Rv position | H37Rv site^1^ | H37Rv re-sequencing^2^ | Locus_tag | Coding product |
| 392190 | c | 390828 | t | c | Rv0323c | hypothetical protein |
| 2061678 | c | 2051746 | t | c | Rv1809 | PPE FAMILY PROTEIN |
| 2730820 | g | 2718852 | t | g |  |  |
| Indel sites in H37Rv validated to be the same as in H37Ra | | | | | | |
| H37Ra coordinates | | H37Rv coordinates | | Variation | Locus_tag | Coding product |
| 132535 | | 131176-131177 | | Insertion |  |  |
| 235857-235858 | | 234496-234497 | | Insertion | Rv0197 | POSSIBLE OXIDOREDUCTASE |
| 425685 | | 424322-424323 | | Insertion | Rv0354c | PPE FAMILY PROTEIN |
| 468809 | | 467500-467501 | | Insertion | Rv0388c | PPE FAMILY PROTEIN |
| 468819 | | 467509-467510 | | Insertion | Rv0388c | PPE FAMILY PROTEIN |
| 1011514 | | 1010206-1010207 | | Insertion | Rv0907 | hypothetical protein Rv0907 |
| 2217491 | | 2207591-2207592 | | Insertion |  |  |
| 2535631-2535632 | | 2525727 | | Deletion | Rv2250A | POSSIBLE FLAVOPROTEIN |
|  |  |  |  |  | Rv2251 | POSSIBLE FLAVOPROTEIN |
| 3591419-3591420 | | 3580637 | | Deletion | Rv3203 upstream | POSSIBLE LIPASE LIPV |
| 3601469 | | 3590686-3590687 | | Insertion |  |  |
| 4409106-4409107 | | 4400661 | | Deletion | Rv3911 | RNA polymerase sigma-70 factor |

1. The published H37Rv genome sequence are retrieved from GenBank (AL123456)

2. The“?”indicates the sites that are not successfully sequenced.
