# Supplementary material for: Genetic Basis of Virulence Attenuation Revealed by Comparative Genomic Analysis of Mycobacterium tuberculosis Strain H37Ra versus H37Rv
Source: PLoS One. 2008 Jun 11;3(6):e2375. doi: 10.1371/journal.pone.0002375 (PMC2440308; doi:10.1371/journal.pone.0002375)
Supplement: Table S5 — (0.05 MB DOC) [file pone.0002375.s006.doc]

**Table S5**. IS elements identified in H37Ra.

| **Family** | **Group** | **Name** | **Complete  Copy** | **Incomplete  copy** |
| --- | --- | --- | --- | --- |
| IS3 | IS51 | IS*6110* | 17 | 1 |
|  |  | IS*1141* |  | 1 |
| IS256 |  | IS*1081* | 5 | 1 |
|  |  | IS*1245* |  | 1 |
|  |  | IS*2606* |  | 1 |
| IS21 |  | IS*Mt2* | 1 |  |
|  |  | IS*Mt3* | 1 | 1 |
| IS5 | IS427 | IS*Mt1* | 2 |  |
| IS607 | IS1535 | IS*1535* | 1 |  |
|  |  | IS*1536* | 1 |  |
|  |  | IS*1537* | 1 |  |
|  |  | IS*1538* | 1 |  |
|  |  | IS*1539* | 1 | 1 |
|  |  | IS*1602* | 1 |  |
| IS110 |  | IS*1547* |  | 2 |
| IS481 |  | IS*Mav2* |  | 1 |
| Total | |  | 32 | 10 |

Note：Thirty two complete IS (insertion sequence) elements, including 17 IS*6110*, belonging to 5 known families (http://www-is.biotoul.fr/is.html) and 10 disrupted IS elements are identified in H37Ra. IS*1547* is known to be a preferential site for IS*6110* insertion [1] and in H37Ra, the only two IS*1547* elements are both interrupted by IS*6110* insertions.

**References:**

1. Fang Z, Doig C, Morrison N, Watt B, Forbes KJ (1999) Characterization of IS1547, a new member of the IS900 family in the Mycobacterium tuberculosis complex, and its association with IS6110. *J Bacteriol* 181: 1021-1024.
