# Supplementary material for: Genetic Basis of Virulence Attenuation Revealed by Comparative Genomic Analysis of Mycobacterium tuberculosis Strain H37Ra versus H37Rv
Source: PLoS One. 2008 Jun 11;3(6):e2375. doi: 10.1371/journal.pone.0002375 (PMC2440308; doi:10.1371/journal.pone.0002375)
Supplement: Table S6 — (0.08 MB DOC) [file pone.0002375.s007.doc]

**Table S6. Variations in the putative promoter regions of H37Ra**

| **Rv position（bp）** | **Variation** | **Locus_tag** | **Rv gene product** | **Upstream of CDS**  **(in H37Ra)** | **Ra**  **coordinate** | **Predicted promoter**  **(in H37Ra)** | **NNPP score**  **(in H37Rv)** |
| --- | --- | --- | --- | --- | --- | --- | --- |
| 42417 | T-C | Rv0039c | transmembrane protein | -66 | MRA_0042 | N | N |
| 131176-131177 | ins G | Rv0108c | hypothetical | -73 | MRA_0114 | -7 ~ -45 | 0.13 |
| 131176-131177 | ins G | Rv0109 | PE-PGRS1 | -206 | MRA_0115 | -43 ~ -83 | 0.21 |
| 459381-459435 | del 55bp | Rv0383c | Secreted protein | -66 | MRA_0391 | N | 0.14 |
| 1123664 | T-A | Rv1005c | para-aminobenzoate  synthase component I | -66 | MRA_1014 | N | N |
| 1123664 | T-A | Rv1006 | hypothetical | -50 | MRA_1015 | -114 ~ -154 | 0.31 |
| 1222797 | G-A | Rv1095 | PhoH-like protein PhoH2 | -200 | MRA_1106 | -155 ~ -195 | 0.12 |
| 1313338 | A-G | Rv1179c | hypothetical | -39 | MRA_1190 | -219 ~ -259 | 0.28 |
| 1313338-1313339 | ins C | Rv1179c | hypothetical | -40 | MRA_1190 | -219 ~ -259 | 0.28 |
| 1561344 | A-G | Rv1386 | PE15 | -120 | MRA_1395 | -70 ~ -110 | 0.81 |
| 2043284 | G-T | Rv1802 | PPE30 | -100 | MRA_1815 | -45 ~ -85 | 0.16 |
| 2167489 | T-C | Rv1917c | PPE34 | -178 | MRA_1928 | -160 ~ -199 | 0.91 |
| 2207591-2207592 | ins C | Rv1964 | integral membrane  protein yrbE3A | -109 | MRA_1975 | -172 ~ -212 | 0.45 |
| 2251999 | A-G | Rv2005c | hypothetical | -116 | MRA_2021 | -19 ~ -59 | 0.19 |
| 2251999 | A-G | Rv2006 | trehalose-6-phosphate phosphatase | -3 | MRA_2022 | -20 ~ -57 | 0.18 |
| 2326893 | A-T | Rv2068c | beta-lactamase | -84 | MRA_2082 | N | N |
| 2326893 | A-T | Rv2069 | RNA polymerase  sigma-70 factor sigC | -51 | MRA_2083 | -26 ~ -66 | 0.19 |
| 2505919 | A-G | Rv2231c | hypothetical | -220 | MRA_2250 | -194 ~ -235 | 0.15 |
| 2718852 | T-G | Rv2421c | nicotinic acid mononucleotide adenyltransferase | -44 | MRA_2447 | -37 ~ -78 | 0.93 |
| 2718852 | T-G | Rv2422 | hypothetical | -231 | MRA_2448 | -123 ~ -163 | 0.89 |
| 3046659 | A-G | Rv2733c | alanine and arginine  rich protein | -136 | MRA_2759 | -10 ~ -50 | 0.26 |
| 3046659 | A-G | Rv2734 | hypothetical | -162 | MRA_2760 | -43 ~ -83 | 0.26 |
| 3133576-3133577 | ins G | Rv2825c | hypothetical | -38 | MRA_2849 | -40 ~ -80 | 0.26 |
| 3415198-3415211 | del 14bp | Rv3053c | glutaredoxin NrdH | -226 | MRA_3085 | -244 ~ -284 | 0.54 |
| 3580637 | del T | Rv3203 | lipase | -1 | MRA_3241 | -112 ~ -152 | 0.28 |
| 3690954-3690955 | ins 58bp | Rv3303c | dihydrolipoamide dehydrogenase | -16 | MRA_3344 | N | N |
| 3690954-3690955 | ins 58bp | Rv3304 | hypothetical | -187 | MRA_3345 | N | N |
| 3862473 | del A | Rv3443c | 50S ribosomal protein L13 | -83 | MRA_3484 | -122 ~ -161 | 0.95 |
| 4052934-4053039 | del 106bp | Rv3610c | cell division protein FtsH | -51 | MRA_3649 | N | 0.10 |
