# Supplementary material for: Genetic Basis of Virulence Attenuation Revealed by Comparative Genomic Analysis of Mycobacterium tuberculosis Strain H37Ra versus H37Rv
Source: PLoS One. 2008 Jun 11;3(6):e2375. doi: 10.1371/journal.pone.0002375 (PMC2440308; doi:10.1371/journal.pone.0002375)
Supplement: Table S7 — (0.07 MB DOC) [file pone.0002375.s008.doc]

**Table S7. Variations in PE/PPE/PGRS family** of H37Ra

| **Ra_locus** | **Rv_Locus** | **Gene Product** | **Variations at aa level (Rv-Ra)_** |
| --- | --- | --- | --- |
| MRA_0115 | Rv0109 | PE_PGRS1 | R346-G |
| MRA_0131 | Rv0124 | PE_PGRS2 | A367-V |
| MRA_0287 | Rv0278c | PE_PGRS3 | R807-G |
| MRA_0288 | Rv0279c | PE_PGRS4 | SNVs 12aa |
| MRA_0363 | Rv0354c | PPE7 | 126fs,184-* |
| MRA_0364 | Rv0355c | PPE8 | W2591-G |
| MRA_0395 | Rv0388c | PPE9 | 7SNVs, 165fs, 444-* |
| MRA_0447 | Rv0442c | PPE10 | K40-E |
| MRA_0539 | Rv0532 | PE_PGRS6 | D227-G, A239-G |
| MRA_0585 | Rv0578c | PE_PGRS7 | E276-G |
| MRA_0754 | Rv0746 | PE_PGRS9 | E191-G, T252-A, N280-D,T320-A,T445-A |
| MRA_0755 | Rv0747 | PE_PGRS10 | R225-G, R227-G K295-R, S300-G |
| MRA_0841a | Rv0833 | PE_PGRS13 | S584-G |
| MRA_0885 | Rv0878c | PPE13 | 436fs, 449-* |
| MRA_0984 | Rv0977 | PE_PGRS16 | A394-S, G404-V |
| MRA_1078 | Rv1068c | PE_PGRS20 | G237-V |
| MRA_1102 | Rv1091 | PE_PGRS22 | G471-S, 501fs, 580-* |
| MRA_1205a | Rv1196 | PPE18 | 308fs, 340-* |
| MRA_1459 | Rv1450c | PE_PGRS27 | S580-I, ins 69aa after 366 |
| MRA_1772 | Rv1759c | WAG22 | 86fs, 117-* |
| MRA_2376 | null | PPE | Inserted |
| MRA_2420 | Rv2396 | PE_PGRS41 | D190-G |
| MRA_2767 | Rv2741 | PE_PGRS47 | S271G |
| MRA_3052 | Rv3021c | PPE47 | 1bp insertion caused frameshift and made Rv3021 and Rv3022 fused into 1 gene in H37Ra |
|  | Rv3022c | PPE48 |  |
| MRA_3177 | Rv3144c | PPE52 | S226G |
| MRA_3384 | Rv3343c | PPE54 | del 576aa (1565-2140) |
| MRA_3391 | Rv3350c | PPE56 | 28 frameshift, 36-* |
| MRA_3407 | Rv3367 | PE_PGRS51 | Ins 3aa after 377 |
| MRA_3428 | Rv3388 | PE_PGRS52 | del 149aa(447-596) |
| MRA_3547 | Rv3507 | PE_PGRS53 | N521H |
| MRA_3548 | Rv3508 | PE_PGRS54 | V1444-A, 1470fs, 1545-* |
| MRA_3551 | Rv3511 | PE_PGRS55 | N396-D, ins 3aa after 638 |
| MRA_3553 | Rv3514 | PE_PGRS57 | SNVs 21aa , del 80aa(654-733), ins 3aa after 847, del 201aa(1055-1255) |
| MRA_3635 | Rv3595c | PE_PGRS59 | 163fs, 239-* |

“fs”: frameshift. “aa”: amino acid. “ins” insert. “del” delete”. * : stop codon.
